# Asparagine-Guided Regulation of Redox Status and Autophagy in Sugar-Starved Lupin (*Lupinus* spp.) Embryonic Axes—A Transcriptomic and Proteomic Approach

**DOI:** 10.3390/ijms27156851

**Published:** 2026-07-30

**Authors:** Szymon Stefaniak, Karolina Wleklik, Katarzyna Nuc, Łukasz Wojtyla, Sławomir Samardakiewicz, Małgorzata Pietrowska-Borek, Ewa Sitkiewicz, Agata Malinowska, Bianka Świderska, Sławomir Borek

**Affiliations:** 1 Department of Plant Physiology, Faculty of Biology, Adam Mickiewicz University Poznań, Uniwersytetu Poznańskiego 6, 61-614 Poznań, Poland; szymon.stefaniak@amu.edu.pl (S.S.); karolina.wleklik@amu.edu.pl (K.W.);; 2 Department of Biochemistry and Biotechnology, Faculty of Agriculture, Horticulture and Biotechnology, Poznań University of Life Sciences, Dojazd 11, 60-632 Poznań, Poland; katarzyna.nuc@up.poznan.pl (K.N.); malgorzata.pietrowska-borek@up.poznan.pl (M.P.-B.); 3 Laboratory of Electron and Confocal Microscopy, Faculty of Biology, Adam Mickiewicz University Poznań, Uniwersytetu Poznańskiego 6, 61-614 Poznań, Poland; slawomir.samardakiewicz@amu.edu.pl; 4 Mass Spectrometry Laboratory, Institute of Biochemistry and Biophysics, Polish Academy of Sciences, Pawińskiego 5a, 02-106 Warsaw, Poland; ewa@ibb.waw.pl (E.S.); esme@ibb.waw.pl (A.M.);

**Keywords:** legume seed germination, carbon limitation, nitrogen metabolism, ATG8, vacuolar hydrolases, peroxisome

## Abstract

Sugar starvation during seed germination requires coordinated regulation of reserve mobilization, redox homeostasis, and intracellular recycling. In lupin seeds, asparagine is a major nitrogen-rich metabolite, but its role in starvation-induced autophagy and redox regulation remains unclear. Here, isolated embryonic axes of white lupin (*Lupinus albus* L.) and Andean lupin (*Lupinus mutabilis* Sweet) were cultured in vitro under sucrose-fed or sugar-starved conditions, with or without asparagine supplementation. Using transcriptomic, proteomic, immunoblot, enzymatic, antioxidant activity, and confocal microscopy analyses, we show that sugar starvation induced redox- and autophagy-related reprogramming, including changes in reactive oxygen species (ROS)-related proteins, catalase accumulation, autophagy-related (*ATG*) gene expression, vacuolar hydrolase-related responses, and proteolytic activity. Peroxisome-associated components, including glycolate oxidase, acyl-CoA oxidase, and catalase, were strongly affected, indicating dynamic remodeling of peroxisome-related metabolism during starvation. Asparagine modified this response by increasing antioxidant capacity and catalase accumulation under sugar starvation, while reducing detectable autophagosome number, many *ATG* and vacuolar hydrolase transcripts, and proteolytic activity. Together with previous evidence for asparagine-induced accumulation of autophagic bodies in vacuoles, these results are consistent with asparagine-dependent modulation of several autophagy-related processes rather than with an effect restricted to a single autophagic step. White and Andean lupin shared the same general regulatory framework but differed in response intensity. Thus, asparagine links nitrogen status with redox stabilization, vacuolar catabolism, and autophagy-related dynamics in sugar-starved lupin embryonic axes.

## 1. Introduction

During seed imbibition and germination, reactive oxygen species (ROS) act both as potentially harmful by-products of resumed metabolism and as redox signals whose levels must be tightly controlled [1,2,3,4]. In germinating lupin seeds, free radicals and H_2_O_2_ accumulate especially in embryonic axes, and carbon starvation further increases free-radical levels [3,4,5,6]. This response is accompanied by activation of antioxidant enzymes; catalase activity increases in embryonic axes and cotyledons of yellow, white, and Andean lupin under carbon-deficient conditions [2,5,7,8,9]. The ascorbate-glutathione system also contributes to redox regulation during lupin germination, helping to maintain ROS within ranges compatible with embryo growth and reserve mobilization [2,9].

Autophagy is a catabolic process that occurs in yeast, plants, and animals, degrading cellular components and recycling metabolites. Macroautophagy, the best-known form of autophagy, begins with the formation of a double-membrane phagophore in the cytoplasm that elongates, encloses the bulk cytoplasm and other cellular components intended for degradation, and transforms into a double-membrane vesicle called an autophagosome. In plant and yeast cells, the autophagosome fuses with the tonoplast via its outer membrane, and the inner vesicle containing the cargo is released into the vacuolar lumen as an autophagic body. The autophagic body is then degraded, and metabolites are recycled [10,11,12]. In plants, the early stages of autophagy, such as phagophore and autophagosome formation, are frequently investigated, whereas the late stages, such as autophagosome degradation and metabolite salvage, are less well studied [11,12]. In contrast, in yeast, the proteins involved in autophagic body degradation have been described since the early 1990s [13]. Knowledge of autophagic body degradation in plant cells remains limited, and only two *Arabidopsis* atypical phospholipases (LCAT3 and LCAT4) have been shown to hydrolyze the autophagic body membrane [14]. It has also been proposed that vacuolar hydrolases, including processing enzymes and lipases, may be associated with autophagic body degradation in plant cells [11,15,16]. After degradation, the permease AVT3 may be involved in metabolite efflux from the vacuole to the cytoplasm [11,17]. The molecular machinery of autophagy is highly conserved across organisms. More than 40 autophagy-related (*ATG*) genes were originally identified in yeast and later in animals and plants. The regulation of this process also shows broad similarities among several plant species, including model species such as *Arabidopsis* and *Nicotiana tabacum*, and crop species such as *Zea mays* and *Oryza sativa* [10]. Many molecular regulators of autophagy have been described, including target of rapamycin (TOR) and sucrose non-fermenting-1-related protein kinase (SnRK1) signaling pathways, phytohormones (e.g., ethylene, salicylic acid, abscisic acid, and brassinosteroids), second messengers (e.g., ROS and Ca^2+^), 26S proteasome and autophagy crosstalk, transcription factors (e.g., WRKY33, HY5, and HsfA1a), and epigenetic regulators (e.g., HDA9) [18]. Under non-stressed conditions, autophagy serves as a basal quality control mechanism by capturing and removing unwanted or defective cellular components, such as organelles and protein complexes [10,11,19]. However, this process intensifies during plant life stages such as germination, reproduction, and senescence [20], as well as under various stress conditions, including carbon starvation [21,22,23,24] or oxidative stress [19,25,26]. In plants, under sugar-starvation conditions, autophagy is required for maintaining cellular metabolic homeostasis [21] and energy status [23], thereby increasing cell survival potential [11,12,27].

During lupin seed germination, complex relationships exist among storage compound mobilization, ROS levels, carbon deficiency, and autophagy. It was shown that in the sucrose-starved isolated embryonic axes of white and Andean lupin, storage lipid breakdown was clearly delayed. Total lipid content remained higher than in sucrose-fed axes, even though the activities of lipase, acyl-CoA oxidase (a source of H_2_O_2_ as a by-product), and catalase were increased. Under the same conditions, sugar-starved axes displayed enhanced autophagy, elevated expression of genes coding for a pexophagy-related machinery, and a reduced amount of the peroxisomal marker Pex14p; importantly, Pex14p abundance increased again when autophagy was inhibited [24]. Additionally, ultrastructural observations of sugar-starved lupin embryo axes revealed structures within autophagic bodies that could be recognized as peroxisomes [22]. On this basis, it was concluded that carbon starvation induces pexophagy in germinating lupin tissues [24]. Because peroxisomes are major sites of both fatty acid β-oxidation and H_2_O_2_ metabolism, and their selective autophagic removal depends on the level of oxidative damage [28,29,30], the degradation of these organelles is expected to affect both ROS production and ROS detoxification. Thus, during lupin seed germination and seedling establishment, autophagy should be regarded not merely as a response to carbon deficit, but also as a mechanism that actively reshapes the redox landscape of the embryonic axis [22,24]. Autophagy can be experimentally retarded by applying synthetic inhibitors such as bafilomycin A1 [31] or concanamycin A [32]. Both compounds are macrolide antibiotics that inhibit the vacuolar-type H^+^-ATPase (V-ATPase), which plays an essential role in maintaining acidic pH within the vacuole [31]. The action of these inhibitors is manifested by the accumulation of autophagic bodies inside the vacuole [33]. A similar accumulation of autophagic bodies was previously observed after treatment with the natural nutrient compound asparagine. We found that this amino acid causes the accumulation of autophagic bodies inside vacuoles during sugar-starvation-induced autophagy in cells of embryonic axes isolated from germinating seeds of yellow, white, and Andean lupin [22,24]. Despite the slowing of degradation of autophagic bodies caused by asparagine in cells of sugar-starved lupin embryonic axes, we observed several symptoms of advanced autophagy, such as strong cell vacuolization [7,22,24,34], decreased phosphatidylcholine levels [24,35] with a concomitant increase in phosphocholine, a metabolic indicator of advanced autophagy [24], and increased transcript abundance of *ATG8*, one of the gene markers of autophagy [24].

The present study continues our previous work on the regulation of metabolism in germinating lupin seeds. Seeds of white lupin (so-called Old-World lupin species) are typically rich in protein and comparatively lower in oil, whereas seeds of Andean lupin (a New-World lupin species) contain both high protein and high lipid levels [36,37,38,39,40,41,42]. Previously, we found that under sugar-starvation conditions, storage lipid mobilization is disrupted by enhanced autophagy, during which peroxisomes (organelles involved in storage lipid breakdown) can be degraded. We also found that asparagine (a key amino acid in lupin seed metabolism) retards autophagic body degradation during starvation-induced autophagy in cells of lupin embryonic axes [22,24]. Given that autophagy is enhanced under starvation conditions and supports cell survival, the observation that asparagine retards autophagic body degradation is intriguing. Moreover, autophagy is part of the cell’s machinery for removing ROS-induced damage or unwanted organelles, such as peroxisomes, thereby contributing to cellular redox homeostasis. Therefore, we combined transcriptomic and proteomic analyses with microscopic observations to characterize the regulatory role of asparagine in the enzymatic oxidative and antioxidative response to starvation in lupin embryonic axes and to define its relationship with autophagy.

## 2. Results

**Figure 1:**
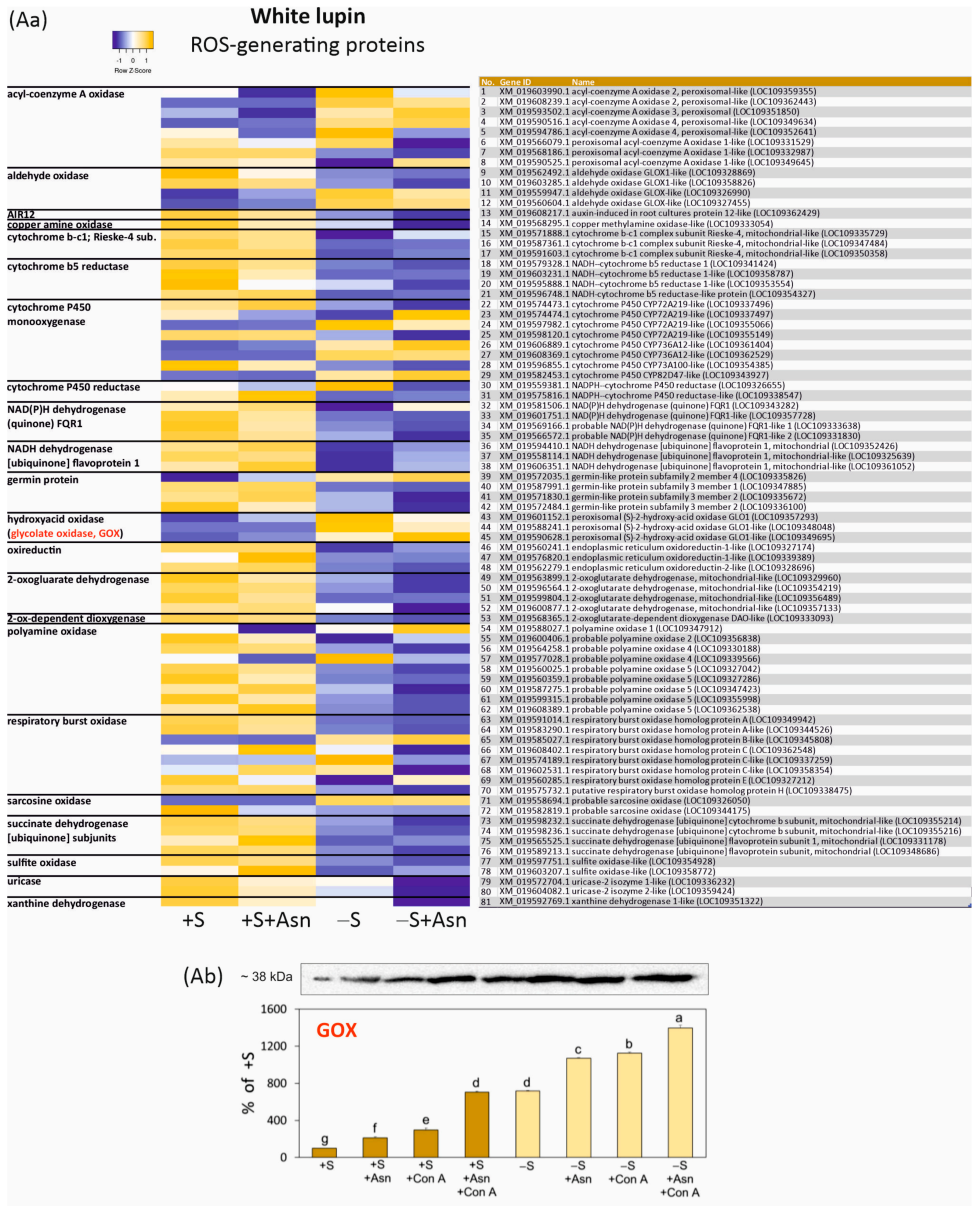

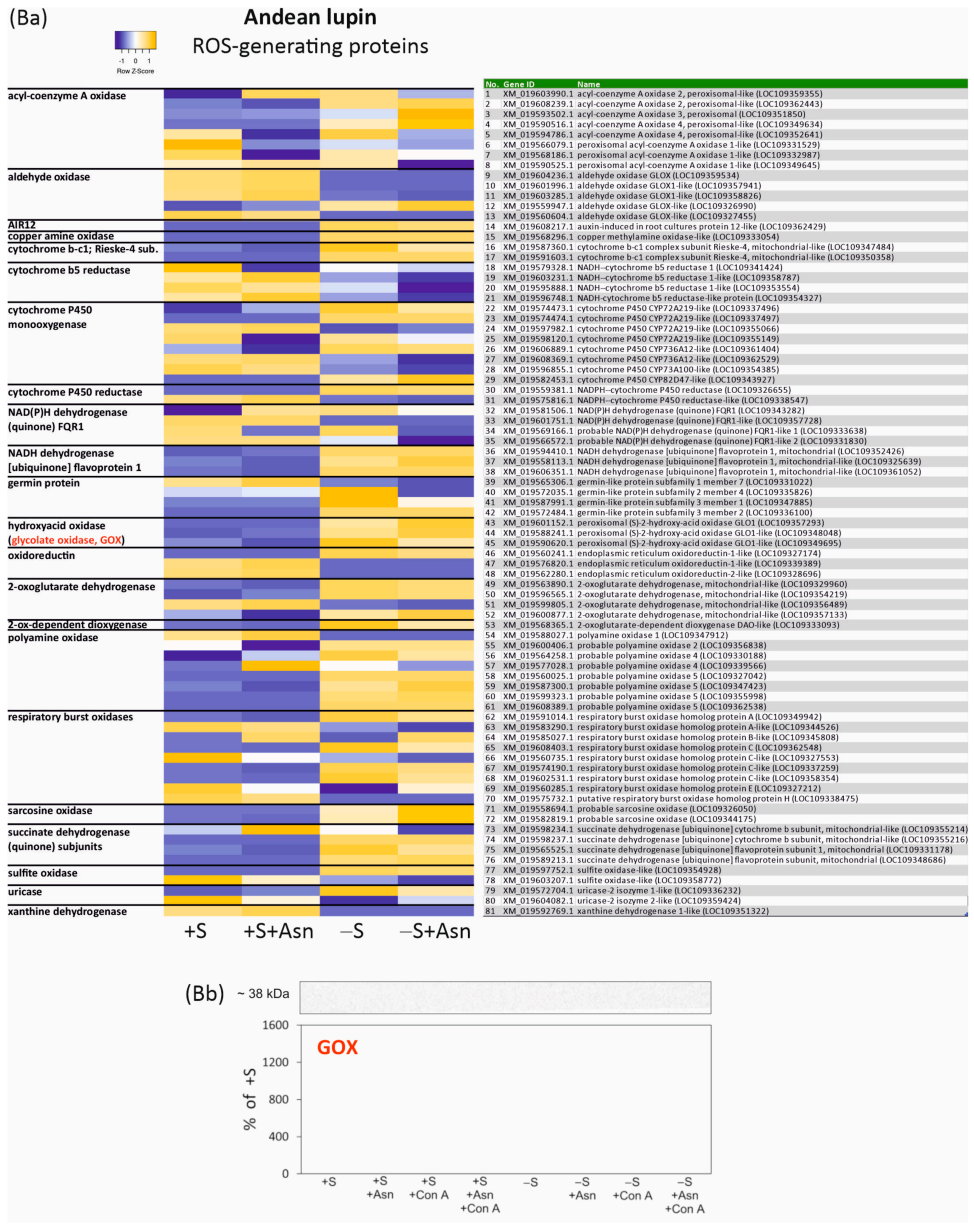
Heatmaps showing relationships among transcript levels of genes encoding reactive oxygen species (ROS)-generating proteins (**Aa**,**Ba**) and a Western blot with densitometric analysis of the glycolate oxidase (GOX) content (**Ab**,**Bb**) in the embryonic axes of white lupin (**A**) and Andean lupin (**B**). Embryonic axes were isolated from germinating seeds and cultured in vitro for 96 h on a medium with 60 mM sucrose (+S) or without the sugar (−S). Media were enriched with 35 mM asparagine (+Asn). The list of transcripts was compiled based on Dumanović et al. [43] and Ali et al. [44]. Data represent averages from three independent experiments, and different letters above the error bars (±SD) indicate statistically significant differences at *p* ≤ 0.05 (ANOVA, Tukey’s HSD multiple-range test). The transcriptomic data used in the heatmap are presented in Supplementary Table S1, and the whole membrane from the representative Western blot is shown in Supplementary Figure S1. In Andean lupin axes, no positive signal was obtained using anti-GOX antibodies.

Previous studies on isolated lupin embryonic axes have demonstrated that carbon starvation leads to approximately a twofold increase in free radicals, including ROS [6]. Therefore, one aim of the present study was to determine how carbon starvation and asparagine affect the transcript levels of genes encoding ROS-generating proteins. Analysis of transcript-level relationships across all four trophic variants of the in vitro culture showed that, in sugar-starved (−S) embryonic axes of white lupin, a general decrease in transcript levels of genes encoding ROS-generating proteins predominated compared with sucrose-fed axes (+S) (Figure 1(Aa)). However, under carbon starvation conditions, transcript levels of most acyl-CoA oxidase genes, all identified hydroxyacid oxidase (glycolate oxidase, GOX) genes, and some respiratory burst oxidase genes increased. In contrast, a markedly different pattern was observed in the embryonic axes of the Andean lupin. In carbon-starved axes (−S) of this species, transcript levels increased for the majority of analyzed genes, including those encoding acyl-CoA oxidase and GOX, relative to sucrose-fed axes (+S) (Figure 1(Ba)). Particular attention was paid to genes encoding acyl-CoA oxidase and GOX because these proteins function in peroxisomes, organelles central to redox homeostasis, and GOX also serves as a peroxisomal marker protein [28,29]. Evaluation of the effect of asparagine on the transcript levels of genes coding for ROS-generating proteins did not show consistent changes in either carbon-starved or sucrose-fed axes of both lupin species (Figure 1(Aa,Ba)). Genes encoding proteins strictly associated with photosynthesis were excluded from the analysis, as the in vitro culture of isolated embryonic axes was conducted in darkness.

iTRAQ-based quantitative proteomic analysis identified six ROS-generating proteins in white lupin axes and four in Andean lupin axes (Table 1). Carbon starvation (−S) in white lupin axes decreased the level of probable NAD(P)H dehydrogenase (quinone) FQR1-like 1 and increased two acyl-coenzyme A oxidase isoforms, two isoforms of peroxisomal (S)-2-hydroxy-acid oxidase GLO1-like (GOX), and quinone oxidoreductase PIG3-like. Asparagine did not cause statistically significant changes in protein content. In Andean lupin axes, similar to white lupin axes, carbon starvation (−S) decreased probable NAD(P)H dehydrogenase (quinone) FQR1-like 1 and peroxisomal acyl-coenzyme A oxidase 3, and increased peroxisomal (S)-2-hydroxy-acid oxidase GLO1-like (GOX) isoforms, and decreased peroxidase 3-like and NADH-cytochrome b5 reductase-like protein. Asparagine caused a statistically significant increase only in NADH-cytochrome b5 reductase-like protein (Table 1).

The transcriptomic (Figure 1(Aa)) and proteomic (Table 1) changes observed for GOX in white lupin embryonic axes were consistent with alterations in GOX protein levels determined by Western blotting. Using anti-GOX antibodies, a statistically significant increase in GOX protein level was detected in −S axes compared with +S axes (Figure 1(Ab)). Asparagine caused a statistically significant increase in GOX protein content in both carbon-starved and sucrose-fed axes (Figure 1(Ab)). Furthermore, a significant increase in GOX levels was observed in response to the autophagy inhibitor concanamycin A, and asparagine further elevated GOX content under both trophic conditions (Figure 1(Ab)). In Andean lupin axes, despite the detection of GOX transcripts and GOX-related peptides by iTRAQ, no positive signal was obtained using anti-GOX antibodies (Figure 1(Bb)). This discrepancy may reflect species-specific differences in antibody epitope recognition, low abundance of the antibody-recognized isoform, differential protein stability or extractability, or iTRAQ detection of GOX-related isoforms not efficiently recognized by immunoblotting.

**Figure 2:**
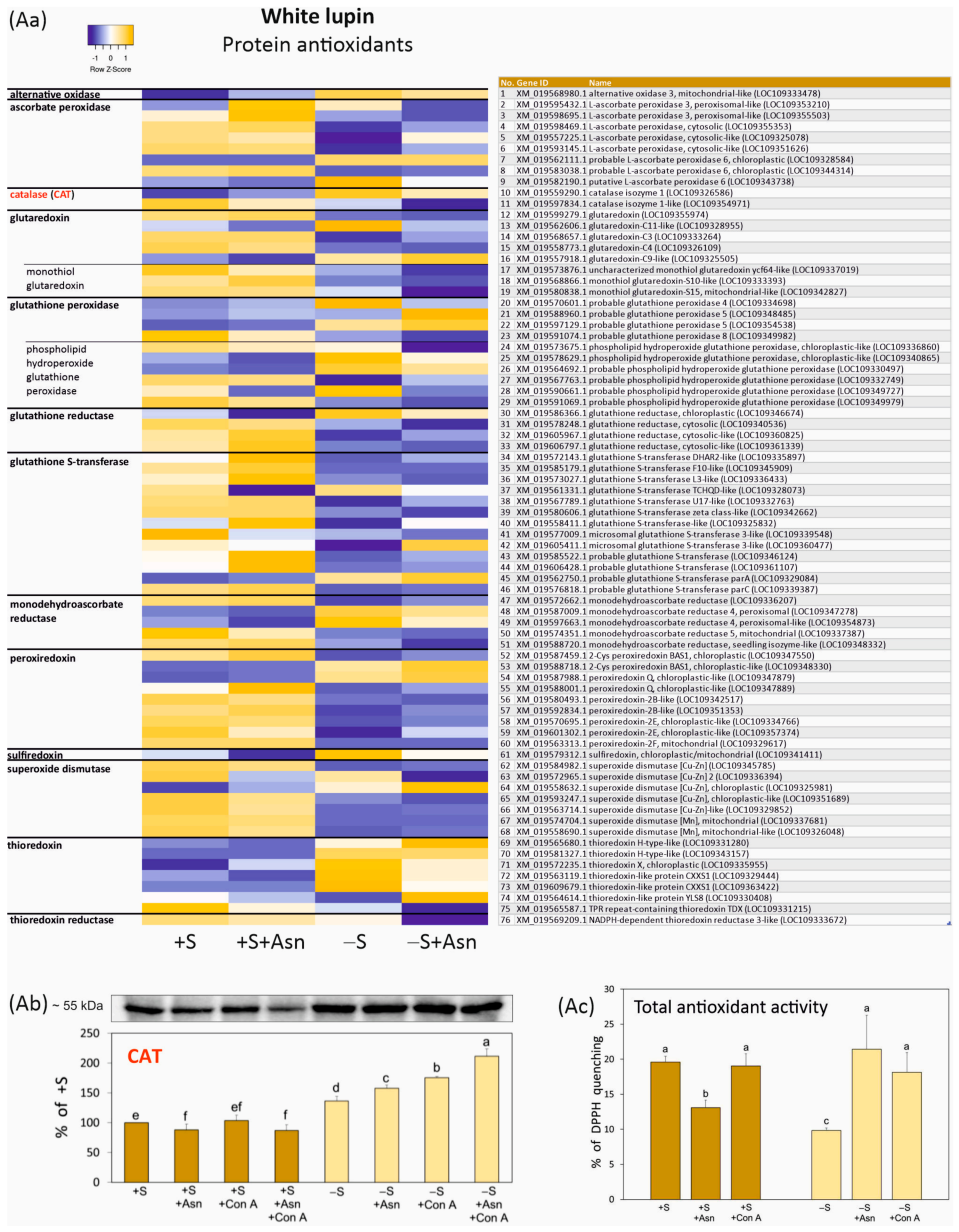

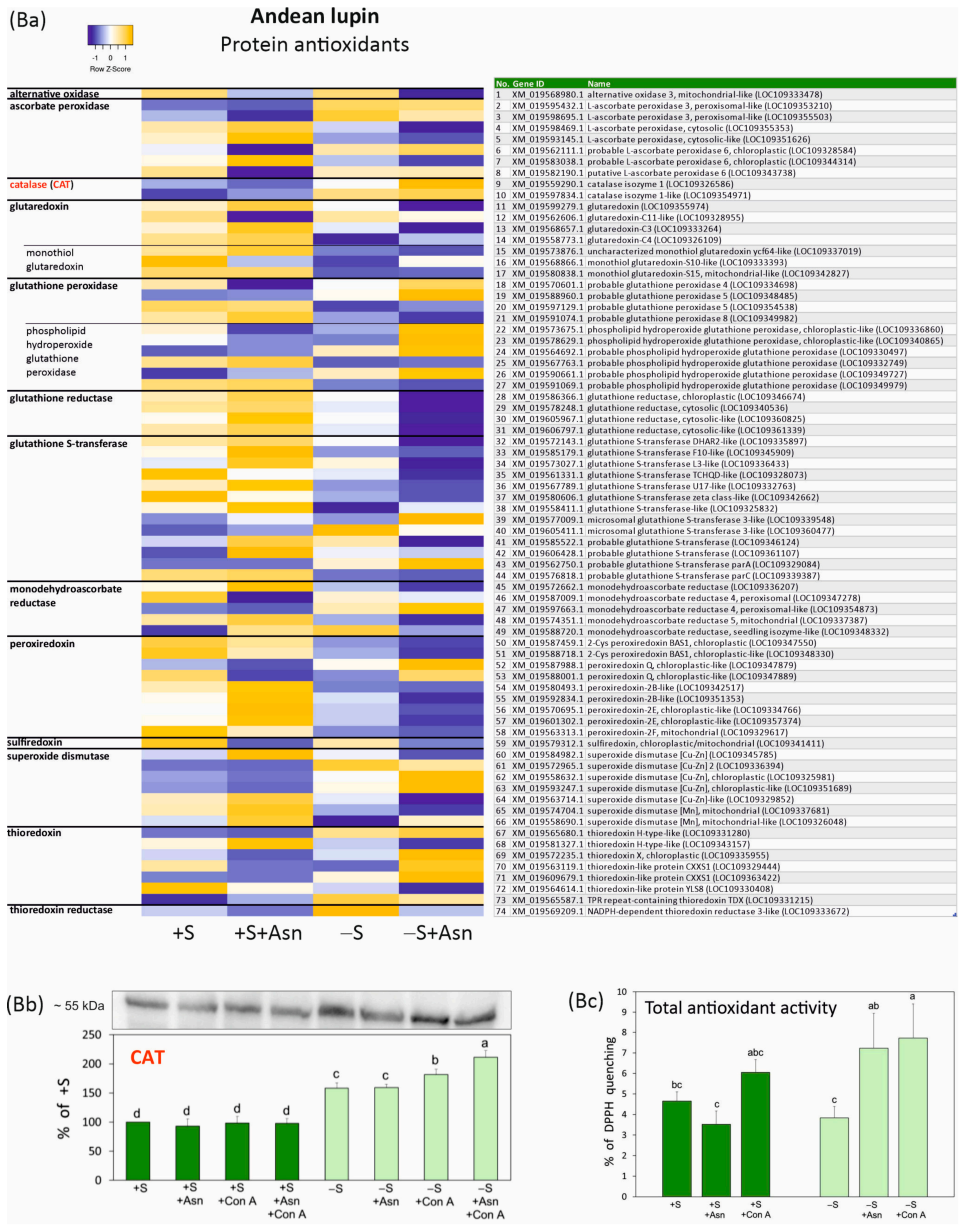
Heatmaps showing relationships among transcript levels of genes encoding protein antioxidants (**Aa**,**Ba**), a Western blot with densitometric analysis of catalase (CAT) content (**Ab**,**Bb**), and total antioxidant activity (**Ac**,**Bc**) in the embryonic axes of white lupin (**A**) and Andean lupin (**B**). Embryonic axes were isolated from germinating seeds and cultured in vitro for 96 h on a medium with 60 mM sucrose (+S) or without the sugar (−S). Media were enriched with 35 mM asparagine (+Asn). The list of transcripts was compiled based on Rajput et al. [47] and Rao et al. [48]. Data represent averages from three independent experiments, and different letters above the error bars (±SD) indicate statistically significant differences at *p* ≤ 0.05 (ANOVA, Tukey’s HSD multiple-range test). The transcriptomic data used in the heatmap are presented in Supplementary Table S2, and the whole membrane from the representative Western blot is shown in Supplementary Figure S1.

During seed germination, enzymatic antioxidant systems are activated as part of redox adjustment [2,3,4,5]. In the present study, we analyzed how in vitro trophic conditions influence transcript levels of genes encoding antioxidant proteins in isolated embryonic axes. In white lupin axes, a clear reduction in transcript levels of many antioxidant enzyme genes was observed in −S axes compared with +S axes. This included genes encoding ascorbate peroxidases, glutathione reductase, glutathione S-transferase, peroxiredoxin, and superoxide dismutase (Figure 2(Aa)). A similar trend was observed in Andean lupin axes (Figure 2(Ba)), although the extent of reduction was less pronounced than in axes of white lupin (Figure 2(Aa)). Regarding the effect of asparagine, both increases and decreases in transcript levels of nearly all identified antioxidant enzyme genes were observed in both sucrose-fed (+S+Asn versus +S) and carbon-starved (−S+Asn versus −S) axes of both species (Figure 2(Aa,Ba)). Notably, the effect of asparagine differed depending on trophic conditions: genes upregulated by asparagine in sucrose-fed axes were typically downregulated in carbon-starved axes. For example, in Andean lupin, asparagine increased the transcript levels of glutathione reductase genes in +S+Asn axes but decreased them in −S+Asn axes (Figure 2(Aa)).

The iTRAQ method identified 14 and 8 antioxidant proteins in the axes of white and Andean lupin, respectively (Table 2). In the axes of white lupin, sugar starvation increased the levels of 9 proteins, mainly related to glutathione metabolism, and decreased 3 proteins, 2 of which are related to ascorbate metabolism. Asparagine significantly increased only 1-Cys peroxiredoxin in the −S white lupin axes. In the axes of Andean lupin, sugar starvation (+S/−S) caused statistically significant decreases in the contents of 4 antioxidant proteins, and asparagine (−S/−S+Asn) significantly increased the catalase isoenzyme 1-like content (Table 2).

Catalase is one of the key antioxidant proteins, whose activity increases during germination and additionally increases under carbon starvation conditions [2,5,7,8,9]. Transcriptomic and proteomic studies, particularly in Andean lupin axes, have shown that catalase transcript levels and protein content are also higher under carbon starvation conditions (Figure 2(Ba) and Table 2). Using specific anti-CAT antibodies and Western blotting, we observed the same relationship, i.e., an increase in catalase content under starvation conditions. Catalase content in −S axes was statistically significantly higher than in +S axes in both studied species (Figure 2(Ab,Bb)), and asparagine additionally increased catalase content in sucrose-starved axes (−S+Asn versus −S), although the asparagine effect was statistically significant only in white lupin axes (Figure 2(Ab)). Furthermore, a clear and statistically significant increase in catalase level caused by an autophagy inhibitor (−S+ConA) was identified in starved axes of both lupin species, and asparagine additionally increased the content of this antioxidant protein under autophagy-limited conditions (−S+Asn+ConA) (Figure 2(Ab,Bb)).

The distinctive effect of asparagine on the antioxidant system was most clearly reflected in total antioxidant activity. This parameter was lower in the −S axes than in the +S axes, and asparagine clearly decreased total antioxidant activity in the +S axes and significantly increased it in the −S axes of both lupin species tested (Figure 2(Ac,Bc)). Concanamycin A had no significant effect on total antioxidant activity in either the +S or −S axes of both lupin species (Figure 2(Ac,Bc)).

**Figure 3:**
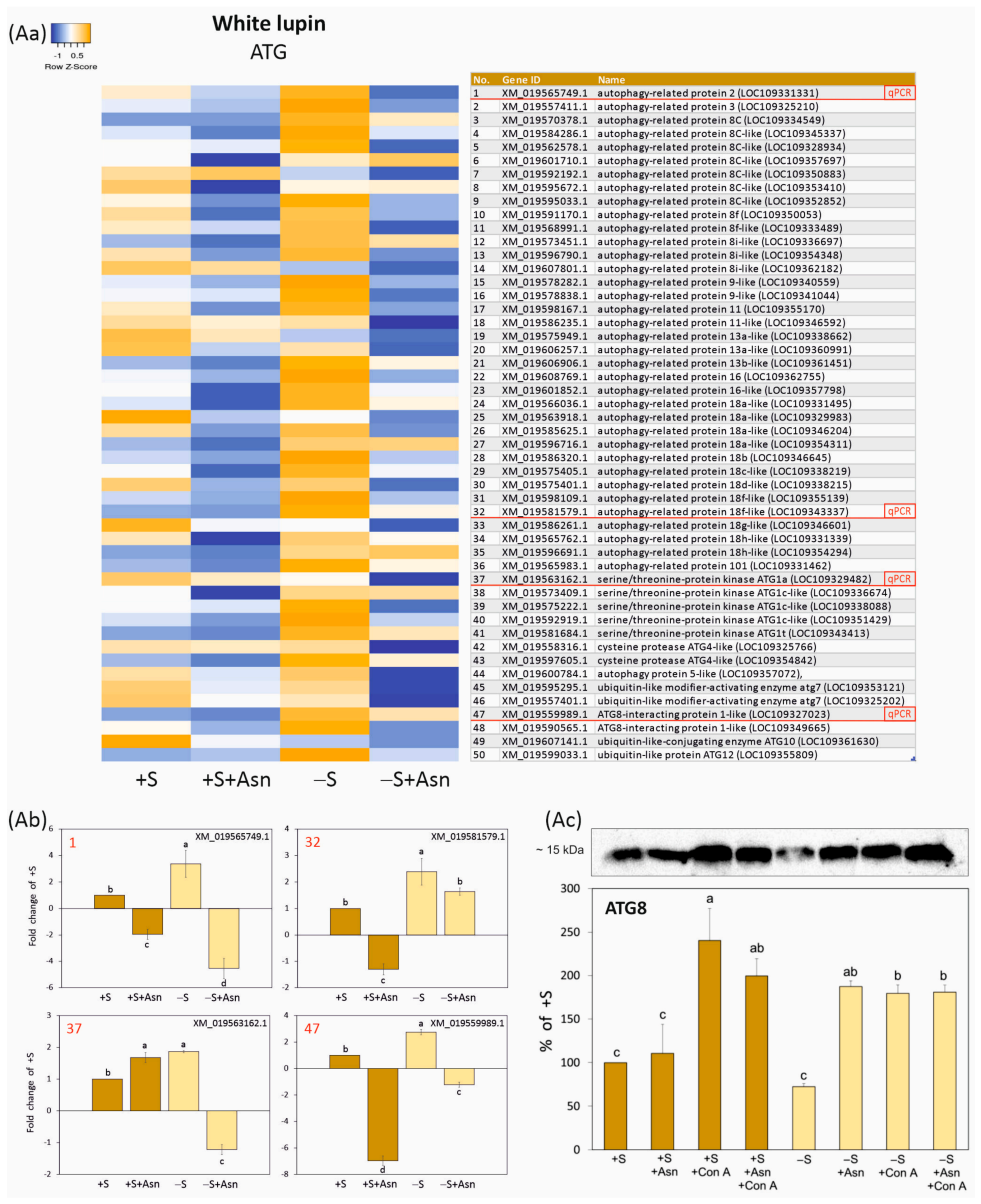

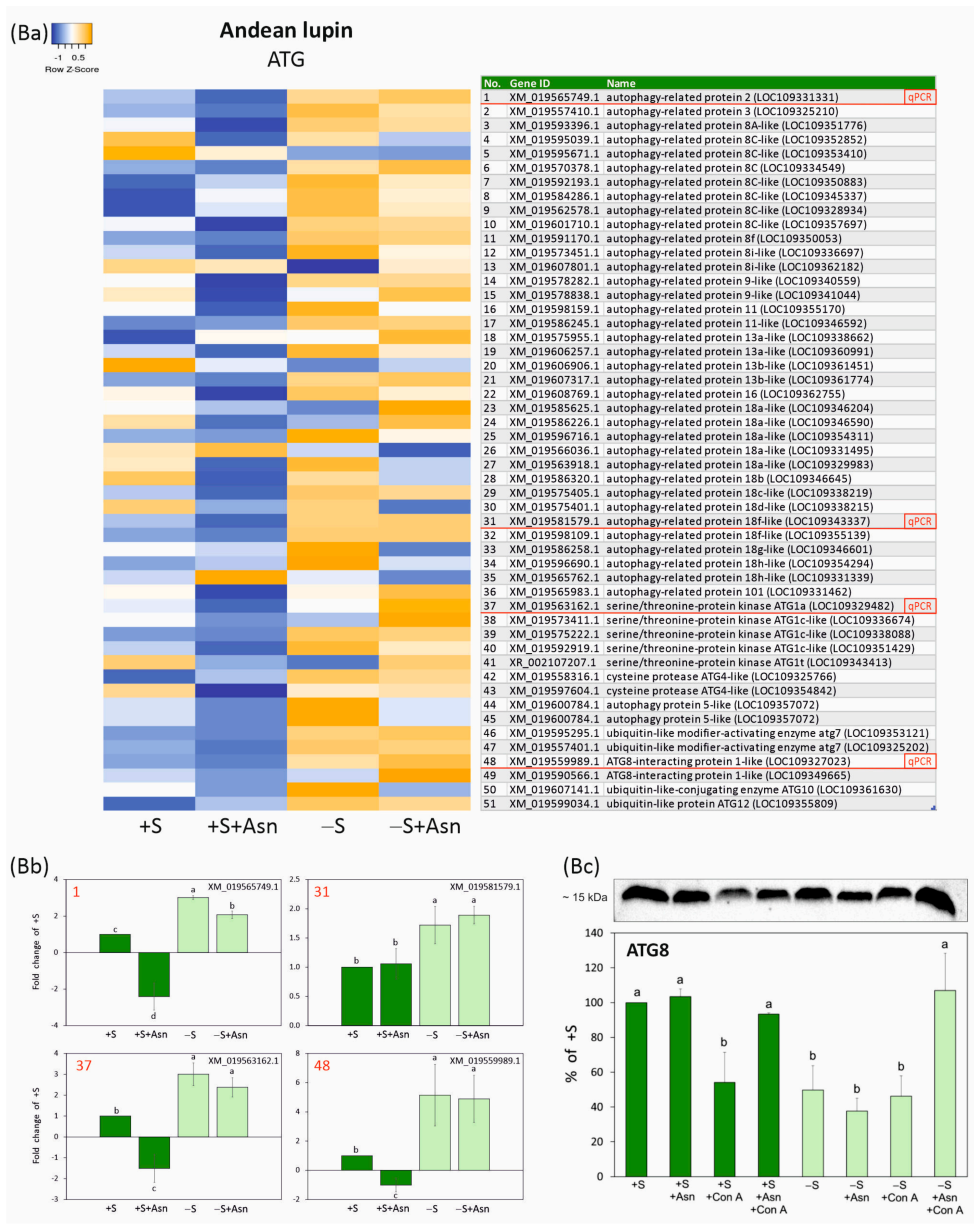
Heatmaps showing relationships among transcript levels of genes encoding autophagy-related (ATG) proteins (**Aa**,**Ba**), relative changes in selected gene transcript levels determined by qRT-PCR (**Ab**,**Bb**), and a Western blot with densitometric analysis of ATG8 content (**Ac**,**Bc**) in the embryonic axes of white lupin (**A**) and Andean lupin (**B**). Embryonic axes were isolated from germinating seeds and cultured in vitro for 96 h on a medium with 60 mM sucrose (+S) or without the sugar (−S). Media were enriched with 35 mM asparagine (+Asn). Data represent averages from three independent experiments, and different letters above or below the error bars (±SD) indicate statistically significant differences at *p* ≤ 0.05 (ANOVA, Tukey’s HSD multiple-range test). The transcriptomic data used in the heatmap are presented in Supplementary Table S3; the primers for qRT-PCR are listed in Supplementary Table S8; and the full membrane from the representative Western blot is shown in Supplementary Figure S1.

Given that autophagic degradation is a key mechanism for removing oxidatively damaged cellular components [19,25,26], transcript levels of *ATG* genes were analyzed. Most *ATG* gene transcripts increased in −S axes compared to +S axes in both species, with a more consistent pattern in white lupin (Figure 3(Aa,Ba)), indicating enhanced autophagy under starvation conditions. Asparagine in sucrose-fed axes clearly reduced the level of the vast majority of *ATG* gene transcripts (+S+Asn versus +S), while in Andean lupin axes, this effect was more pronounced. However, a clear reduction in the level of gene transcripts under starvation conditions (−S+Asn versus −S) was observed only in white lupin axes (Figure 3(Aa,Ba)). The changes in the content of *ATG* gene transcripts observed in the NGS results were selectively confirmed by RT-qPCR (Figure 3(Ab,Bb)).

ATG8 is a widely used marker protein of autophagy [49]; therefore, using specific anti-ATG8 antibodies and Western blotting, we determined changes in ATG8 content in the axes of both lupin species. In white lupin axes, ATG8 content was similar in the +S and −S axes, and asparagine significantly increased ATG8 content only in the −S axes. However, an autophagy inhibitor (concanamycin A) clearly increased ATG8 content in both the +S and −S axes (Figure 3(Ac)). In contrast to white lupin axes, carbon starvation in Andean lupin axes caused a significant decrease in ATG8 content (Figure 3(Bc)), which may be consistent with increased delivery and degradation of ATG8 in the vacuole during active autophagy [11,49], although alternative explanations related to ATG8 turnover or accumulation cannot be excluded. Asparagine did not cause statistically significant changes in ATG8 content in either +S or −S axes, while concanamycin A decreased ATG8 content in +S axes. However, under starvation conditions, asparagine, together with an autophagy inhibitor, significantly increased ATG8 content (−S+Asn+ConA) (Figure 3(Bc)).

**Figure 4:**
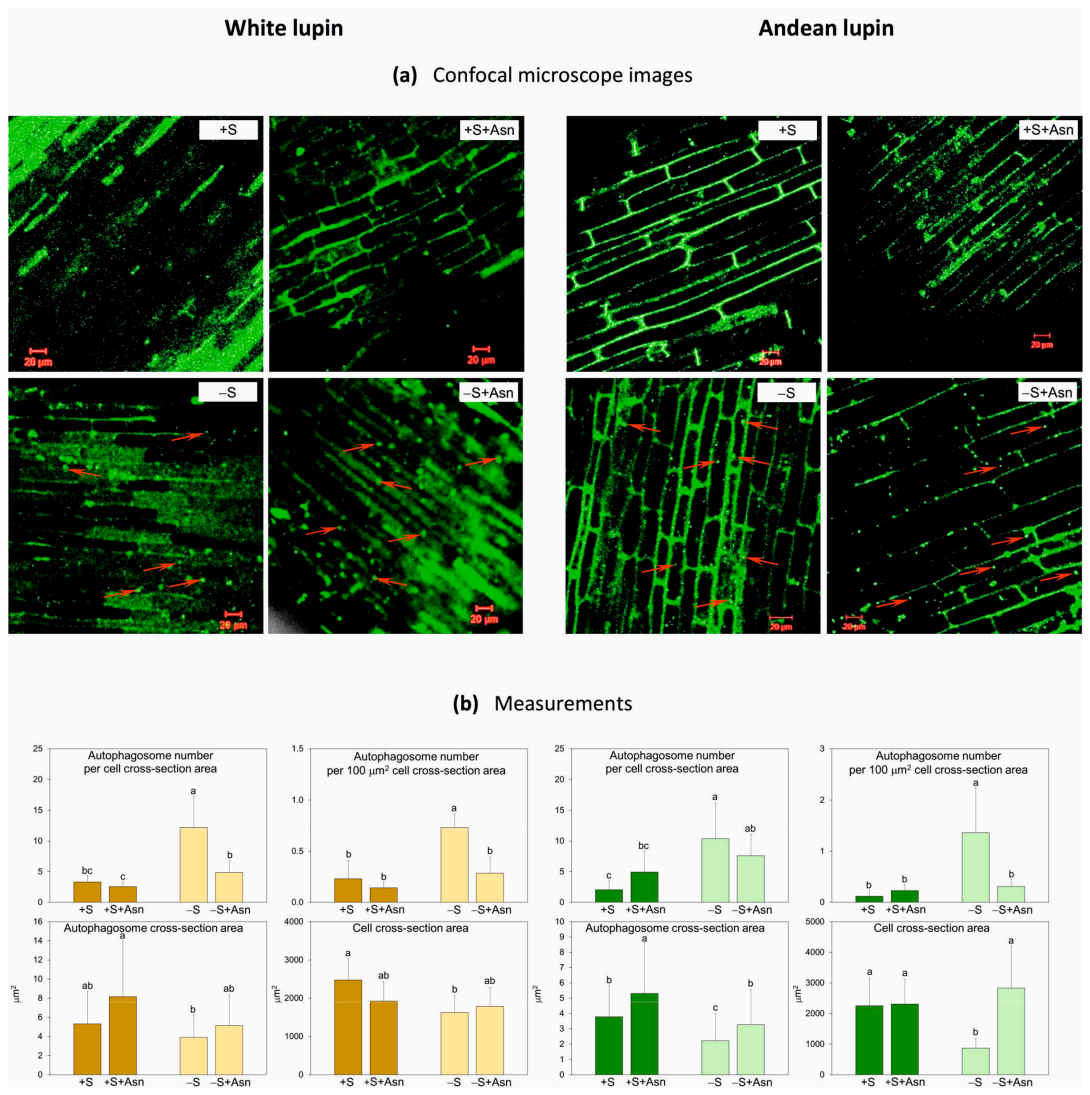
Representative confocal microscopy images showing autophagosomes stained with monodansylcadaverine ((**a**); red arrows) and selected autophagosome and cell parameters (**b**) in root tips of white and Andean lupin embryonic axes. Embryonic axes were isolated from germinating seeds and cultured in vitro for 96 h on a medium with 60 mM sucrose (+S) or without sucrose (−S). Media were enriched with 35 mM asparagine (+Asn). Data represent averages from three independent experiments, and different letters above the error bars (±SD) indicate statistically significant differences at *p* ≤ 0.05 (ANOVA, Tukey’s HSD multiple-range test).

Our previous observations of the ultrastructure of cells in the root meristematic zone of lupin embryonic axes have shown a clear slowdown in the degradation of autophagic bodies in the vacuole under the influence of asparagine [22,24]. In the present study, confocal microscopy showed that asparagine also significantly reduced the number of autophagosomes in root-tip cells of axes of both lupin species (Figure 4).

**Figure 5:**
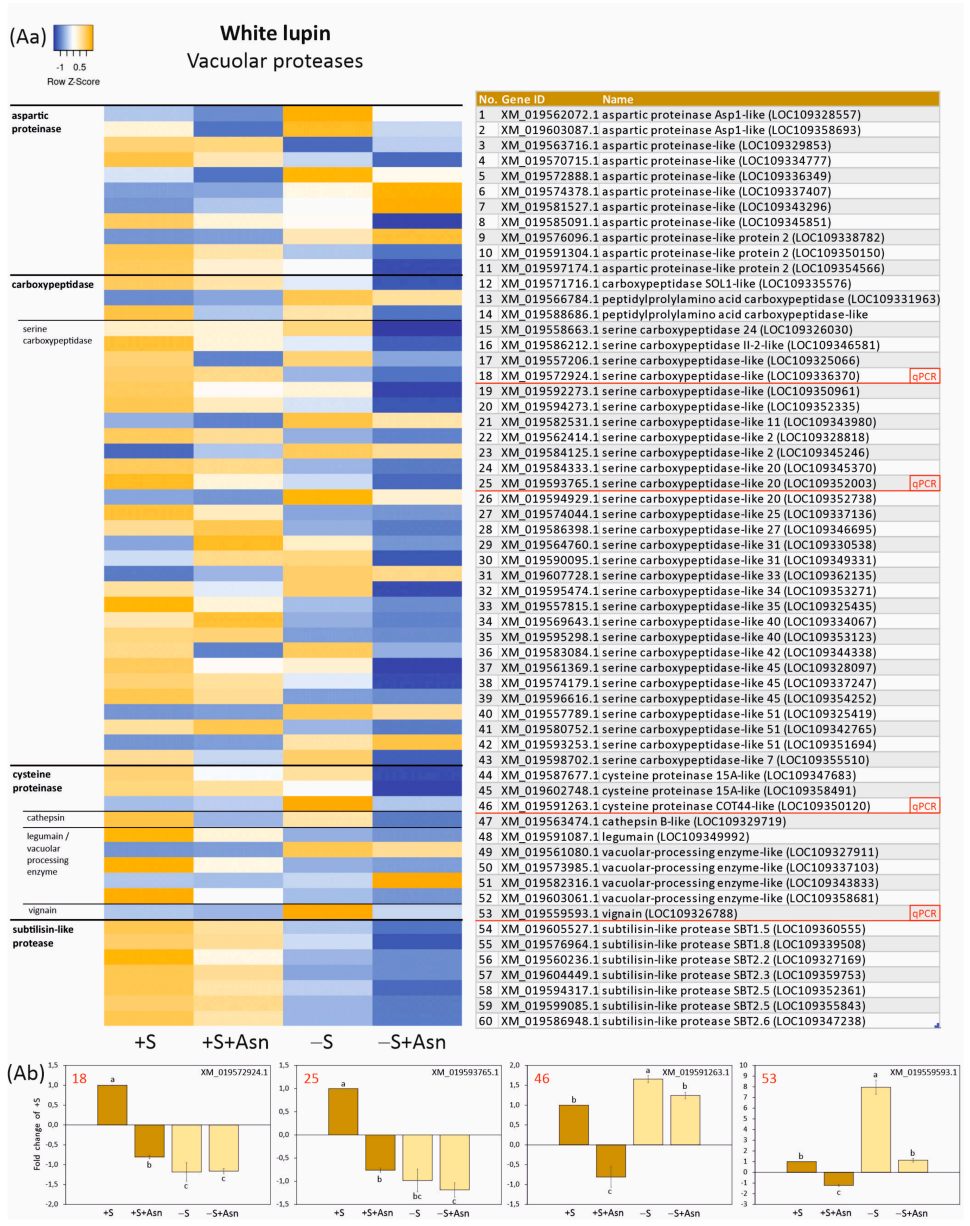

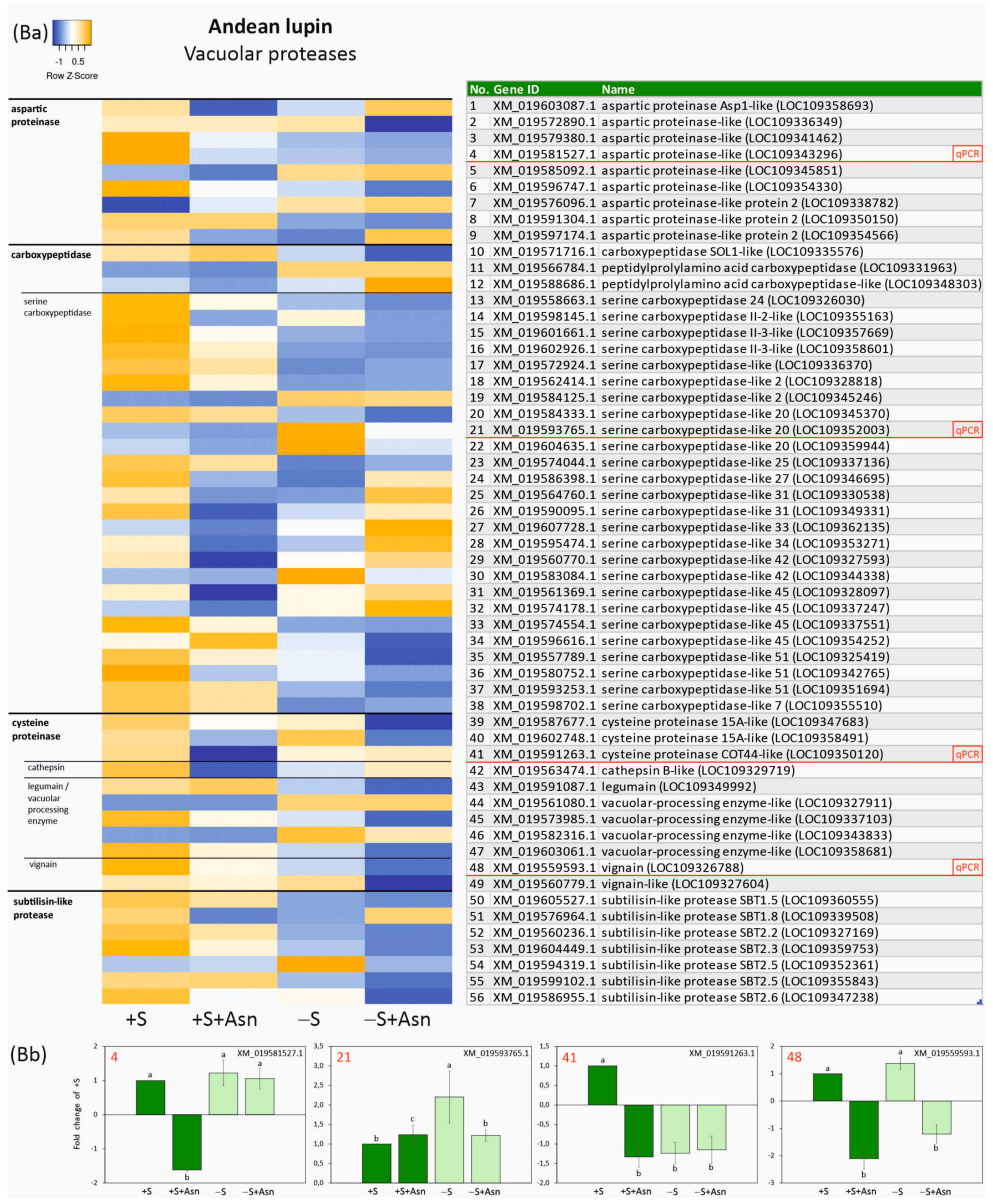
Heatmaps showing relationships among transcript levels of genes encoding predicted vacuolar proteases (**Aa**,**Ba**) and relative changes in selected gene transcript levels determined by qRT-PCR (**Ab**,**Bb**) in the embryonic axes of white lupin (**A**) and Andean lupin (**B**). Embryonic axes were isolated from germinating seeds and cultured in vitro for 96 h on a medium with 60 mM sucrose (+S) or without the sugar (−S). Media were enriched with 35 mM asparagine (+Asn). The list of transcripts was generated using DeepLoc 2.0 software (https://services.healthtech.dtu.dk/services/DeepLoc-2.0/ (accessed on 11 March 2025)) with a high-quality model (Supplementary Table S4). Data represent averages from three independent experiments, and different letters above or below the error bars (±SD) indicate statistically significant differences at *p* ≤ 0.05 (ANOVA, Tukey’s HSD multiple-range test). The transcriptomic data used in the heatmap are presented in Supplementary Table S5, and the primers for qRT-PCR are listed in Supplementary Table S8.

**Figure 6:**
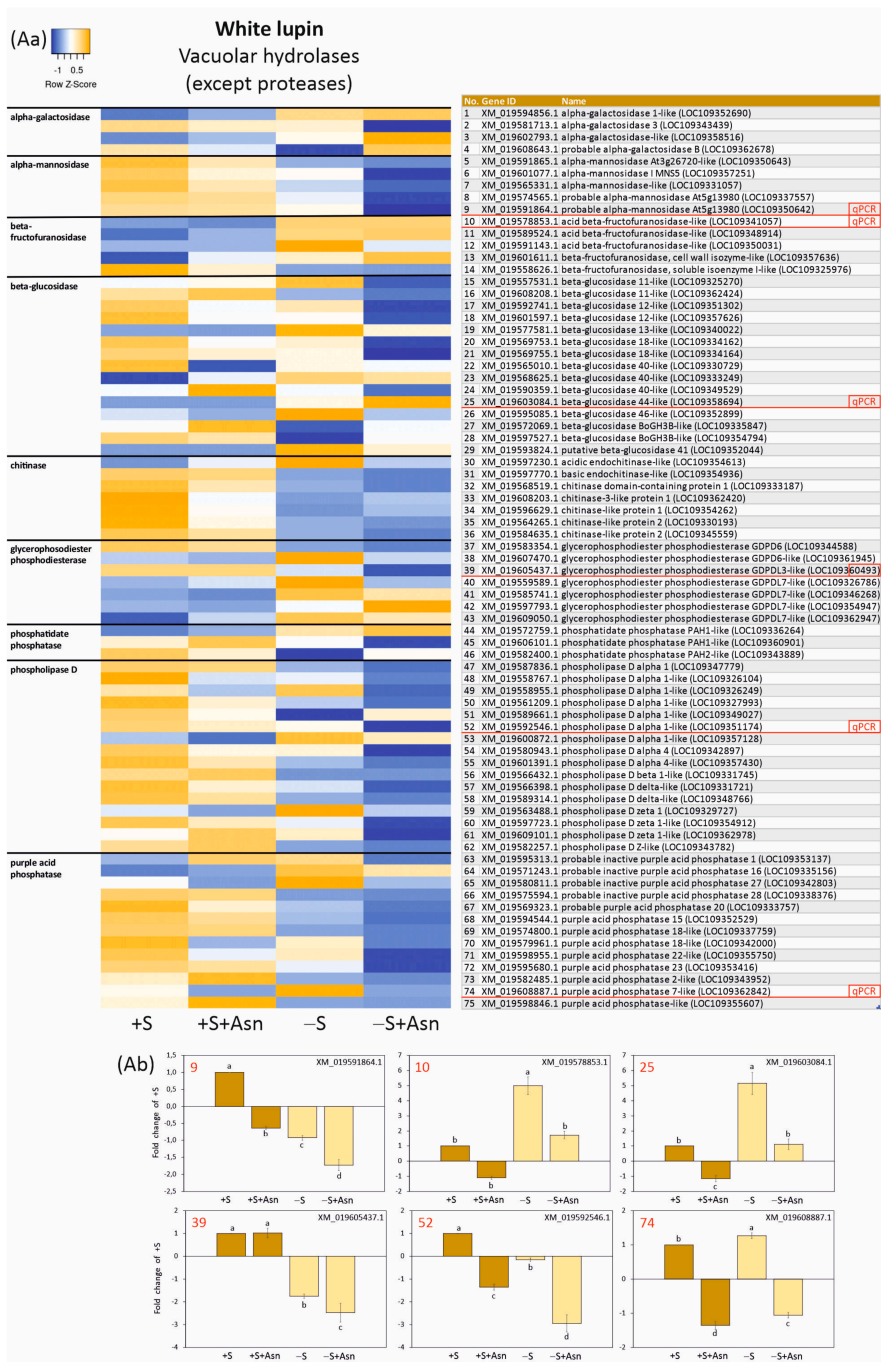

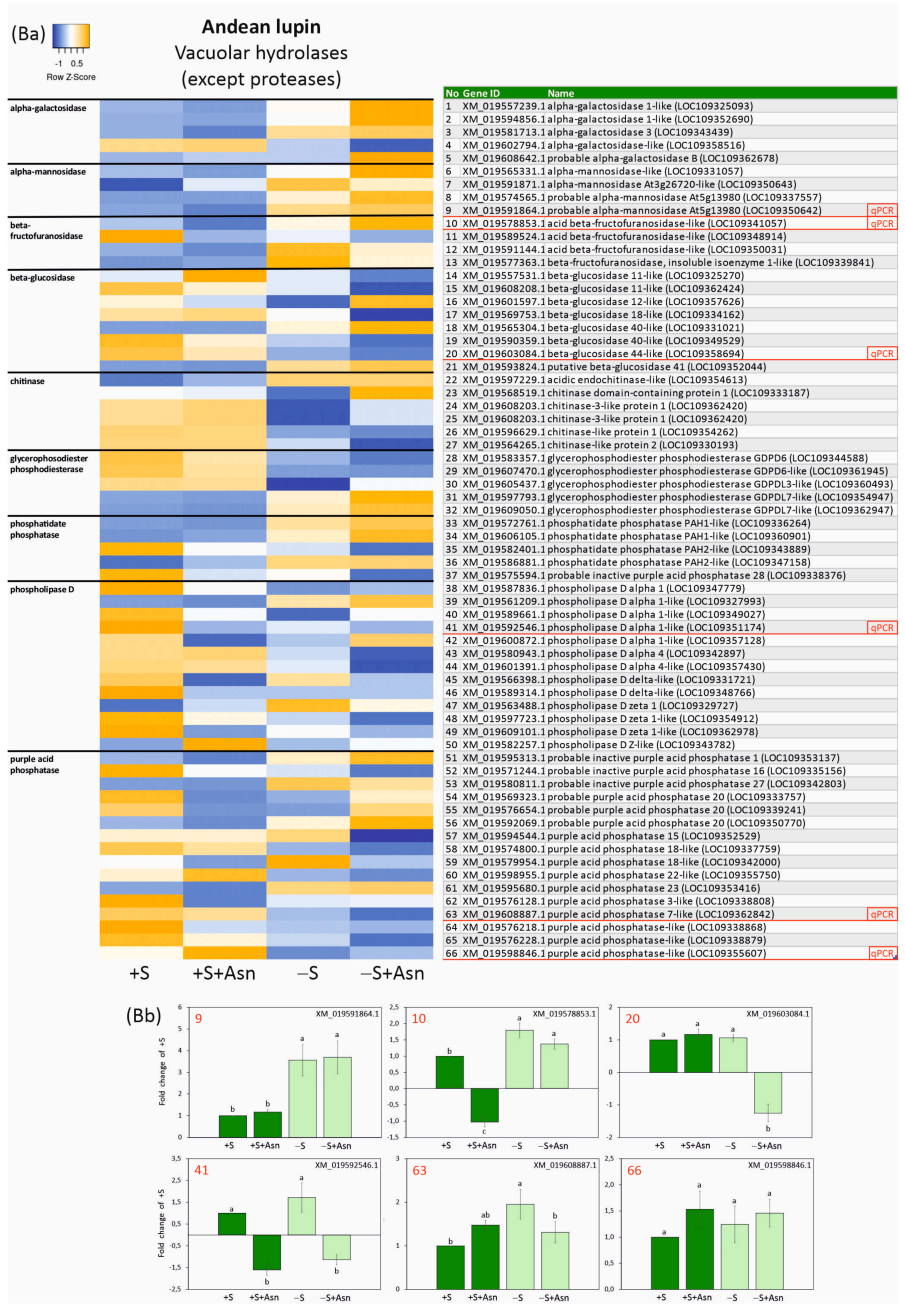
Heatmaps showing relationships among transcript levels of genes encoding predicted vacuolar hydrolases ((**Aa**,**Ba**); with the exception of predicted vacuolar proteases) and relative changes in selected gene transcript levels determined by qRT-PCR (**Ab**,**Bb**) in the embryonic axes of white lupin (**A**) and Andean lupin (**B**). Embryonic axes were isolated from germinating seeds and cultured in vitro for 96 h on a medium with 60 mM sucrose (+S) or without the sugar (−S). Media were enriched with 35 mM asparagine (+Asn). The list of transcripts was generated using DeepLoc 2.0 software (https://services.healthtech.dtu.dk/services/DeepLoc-2.0/ (accessed on 11 March 2025)) with a high-quality model (Supplementary Table S6). Data represent averages from three independent experiments, and different letters above or below the error bars (±SD) indicate statistically significant differences at *p* ≤ 0.05 (ANOVA, Tukey’s HSD multiple-range test). The transcriptomic data used in the heatmap are presented in Supplementary Table S7, and the primers for qRT-PCR are listed in Supplementary Table S8.

As mentioned above, our previous studies have shown that asparagine in cells of starved lupin embryonic axes slows the degradation of autophagic bodies and causes their accumulation in vacuoles [22,24]. As vacuolar hydrolytic enzymes are responsible for the degradation of autophagic bodies, we first examined how trophic conditions in the in vitro culture affect the interrelationships among transcript levels of genes encoding predicted vacuolar proteases. These enzymes are believed to be crucial for the degradation of autophagic bodies; however, this assumption is based mainly on studies conducted in yeast, which confirmed the involvement of proteinase A and proteinase B in this process [11,13]. Our results showed that the transcript levels of many genes encoding vacuolar proteases were clearly reduced by asparagine under starvation conditions (−S+Asn versus −S) (Figure 5(Aa,Ba)), and this effect was more pronounced in white lupin axes. Among the vacuolar protease genes whose transcript levels were reduced by asparagine were serine carboxypeptidases, cysteine proteinases, some vacuolar processing enzyme (legumins) genes, and subtilisin-like proteases (Figure 5(Aa,Ba)). A separate analysis was performed for predicted vacuolar hydrolases other than proteases (Figure 6A,B). Lipases were excluded from the present hydrolase dataset because these enzymes were analyzed in detail in a separate study [15]. In the present study, in −S+Asn white lupin axes compared with −S axes (−S+Asn versus −S), we found reduced transcript abundance of many genes, including alpha-mannosidases, beta-glucosidases, chitinases, phospholipase D, and purple acid phosphatases (Figure 6(Aa)). In Andean lupin axes, the effect of asparagine was less consistent than in white lupin axes, as this amino acid increased the transcript levels of some genes and decreased others (Figure 6(Ba)). To confirm the NGS results for selected genes, quantitative RT-qPCR analyses were performed (Figure 5(Ab,Bb) and Figure 6(Ab,Bb)).

iTRAQ-based quantitative proteomic analysis identified 13 hydrolases in white lupin axes and 6 in Andean lupin axes, of which 8 and 5 were vacuolar hydrolases in white and Andean lupin axes, respectively (Table 3). Among the vacuolar hydrolases, carbon starvation (+S/−S) in white lupin axes led to an increase in the content of some enzymes (e.g., probable alpha-mannosidase, proline carboxypeptidase, and cysteine proteinase COT44-like) and a decrease in the content of others (e.g., phospholipase D alpha 1-like, subtilisin-like protease SBT1.5, and subtilisin-like protease SBT2.5). Among the vacuolar hydrolases in Andean lupin axes, an increase in the content of cathepsin B-like and a decrease in the content of bifunctional purple acid phosphatase 26 were observed (Table 3). Asparagine under starvation conditions (−S/−S+Asn) reduced the content of only cysteine proteinase COT44-like in white lupin axes and of phospholipase D alpha 1 in Andean lupin axes (Table 3). All changes were statistically significant at q < 0.05.

**Figure 7:**
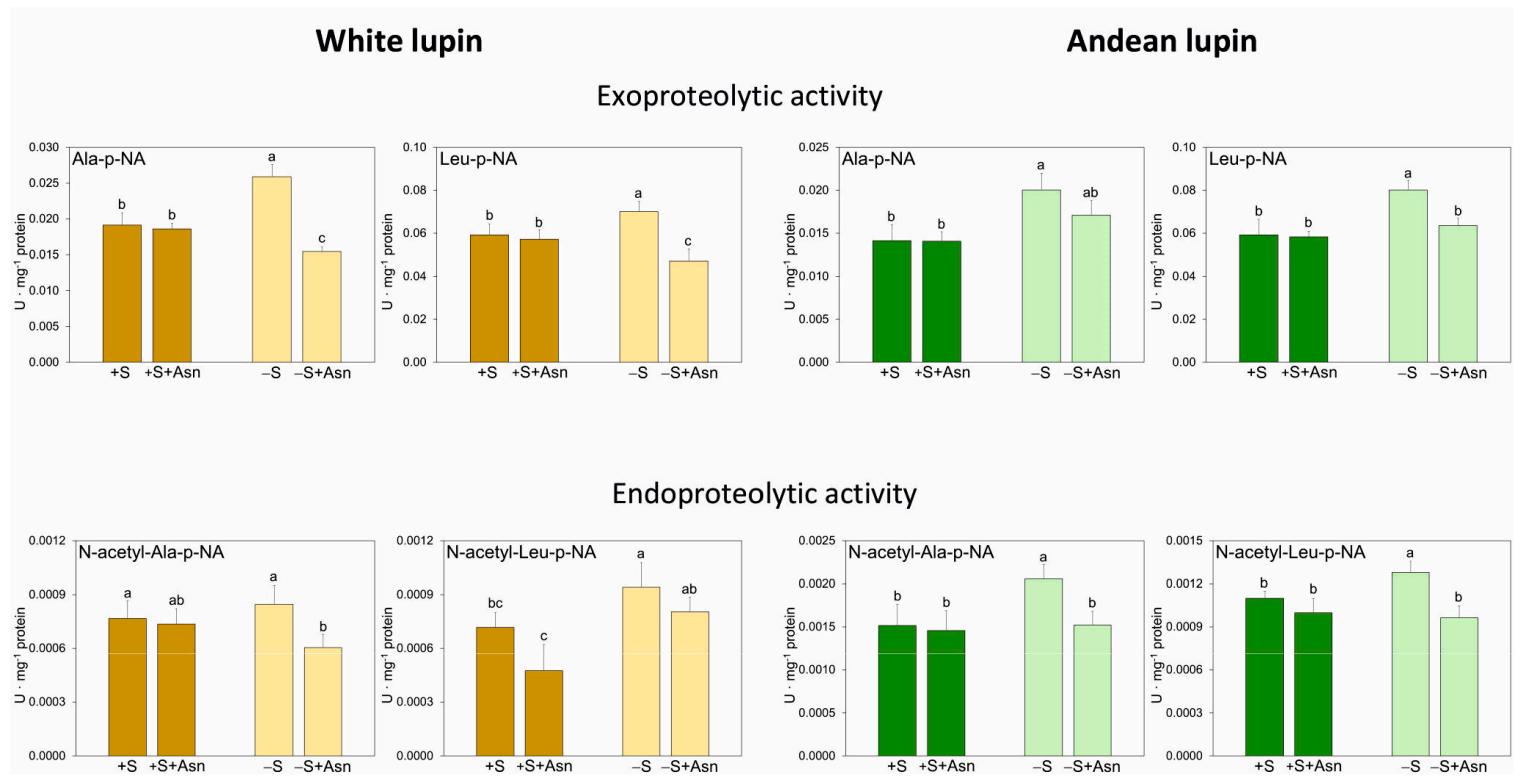
Total exo- and endoproteolytic activity in the embryonic axes of white and Andean lupin. Embryonic axes were isolated from germinating seeds and cultured in vitro for 96 h on a medium with 60 mM sucrose (+S) or without the sugar (−S). Media were enriched with 35 mM asparagine (+Asn). For specific exo- and endoproteolytic activity, nitroanilide substrates were used. Data represent averages from three independent experiments, and different letters above the error bars (±SD) indicate statistically significant differences at *p* ≤ 0.05 (ANOVA, Tukey’s HSD multiple-range test).

We also examined how carbon starvation and asparagine affect proteolytic activity in the embryonic axes of both lupin species. Using specific substrates for exo- and endopeptidases, we found that sugar starvation significantly elevated exo- and endoproteolytic activity in axes of both lupin species (Figure 7). Asparagine did not alter proteolytic activity in the sucrose-fed axes, and the observed differences were not statistically significant. In contrast, in starved axes of both lupin species, asparagine clearly reduced the activity of both exo- and endopeptidases (Figure 7).

## 3. Discussion

### 3.1. Sugar Starvation Remodels Redox and Peroxisome-Related Metabolism

The present study shows that sugar starvation in the embryonic axes of white and Andean lupin triggers not merely a stress response but broad metabolic reprogramming in which redox homeostasis, autophagy, and vacuolar hydrolysis are tightly interconnected. In both species, carbon deprivation was associated with enhanced expression of many *ATG* genes (Figure 3A,B), increased proteolytic activity (Figure 7), altered abundance of ROS-related enzymes (Table 1 and Table 2), and a decline in total antioxidant activity (Figure 2(Ac,Bc)), indicating that the sugar-starved embryonic axis enters a catabolic, survival-oriented state. These observations are consistent with the established role of autophagy as a core acclimatory mechanism during nutrient limitation, especially under carbon starvation, when plant cells must recycle intracellular constituents to sustain energy balance and metabolic homeostasis [21,23,50,51].

Our data extend this framework by showing that, in lupin embryonic axes, the starvation response is closely associated with adjustments in the redox network. Under sugar starvation, several components of peroxisome-related ROS metabolism were affected, as indicated by the increased abundance of GOX detected by Western blot in white lupin axes (Figure 1(Ab)), changes in GOX-related proteins and selected acyl-CoA oxidase isoforms identified by iTRAQ (Table 1), and the starvation-induced increase in catalase content in both lupin species (Figure 2(Ab,Bb)). At first glance, the maintenance or increase in selected peroxisome-associated enzymes may appear difficult to reconcile with our previous evidence that sugar starvation induces pexophagy in lupin embryonic axes [24]. However, these observations are not necessarily contradictory. Pexophagy should be interpreted here as enhanced peroxisome turnover and functional remodeling of the peroxisomal compartment rather than as simple bulk depletion of all peroxisomes or all peroxisomal proteins. Thus, under sugar starvation, damaged or metabolically redundant peroxisomes may be selectively removed, while the remaining or newly formed peroxisomal machinery is adjusted to support lipid-related metabolism and ROS homeostasis. In this context, the previously observed decrease in the peroxisomal marker Pex14p under starvation and its accumulation after autophagy inhibition can be reconciled with the present changes in GOX, acyl-CoA oxidase isoforms, and catalase as evidence for dynamic remodeling, rather than simple disappearance, of the peroxisomal compartment. More generally, these results are consistent with the view that carbon starvation remodels peroxisome function and turnover as part of a broader reconfiguration of the redox landscape of the embryonic axis. Recent reviews further emphasize that ROS and autophagy should be viewed as mutually coupled adaptive processes rather than independent stress outputs, with ROS acting both as damaging agents and as signals that modulate autophagic flux [25,28,30,44,50]. These results are also consistent with the broader concept that stress resilience in crops depends on precise regulation of ROS-related responses and stress-adaptive metabolic processes [52].

Against this background, the role of asparagine emerges as particularly important. In lupin, asparagine is not simply one amino acid among many but a central metabolite of seed nitrogen metabolism, a major transport and storage form of nitrogen, and a prominent product of reserve mobilization during germination [53,54]. In darkened or carbon-limited tissues of several plant species, including roots, asparagine synthetase expression is induced while asparagine accumulates as a major sink for nitrogen released by proteolysis, reflecting a metabolic shift toward a high N:C state [55,56,57,58]. In lupin specifically, asparagine may account for a very large fraction of seedling dry mass during the transient post-germinative phase, before nitrogen-fixing symbiosis is fully established [54], and it also promotes starch accumulation while decreasing soluble sugar levels [59]. Therefore, under the conditions used here, exogenous asparagine should be considered not only as an additional nitrogen source but also as a metabolite that intensifies the imbalance between nitrogen availability and carbon shortage.

### 3.2. Asparagine Modulates Autophagy-Related Dynamics and Vacuolar Hydrolysis

This perspective helps to explain the apparently paradoxical effects of asparagine observed here and in our earlier studies. Carbon starvation enhances autophagy in cells of lupin embryonic axes [7,22,24,34], a response generally considered beneficial for survival under energy deficit. However, asparagine modifies this starvation-induced autophagic response in a more complex way than simple stimulation or inhibition of the pathway. In starved embryonic axes, asparagine retards the degradation of autophagic bodies and causes their accumulation in vacuoles [22,24], but at the same time reduces the number of autophagosomes detectable by MDC staining (Figure 4), decreases the transcript levels of many *ATG* genes, especially in white lupin (Figure 3A), lowers the transcript abundance of numerous predicted vacuolar proteases (Figure 5A,B) and other vacuolar hydrolases (Figure 6A,B), and suppresses overall exo- and endoproteolytic activity (Figure 7). This interpretation is further supported by our separate analysis of predicted vacuolar lipases, which were excluded from the present hydrolase dataset because they were analyzed in detail elsewhere [15]. In that study, asparagine also caused a clear decrease in the transcript levels of many lipase genes in embryonic axes of both lupin species, further supporting the interpretation that this amino acid is associated with reduced vacuolar hydrolytic capacity during starvation-induced autophagy. Taken together, these observations indicate that asparagine does not act exclusively at a single step of the pathway and does not simply suppress autophagy. Rather, the combined data suggest that it reshapes autophagy-related dynamics across multiple levels, combining altered autophagosome dynamics or steady-state autophagosome abundance with reduced vacuolar hydrolytic capacity and delayed degradation of autophagic bodies.

The present results also highlight an important methodological point: autophagosome abundance should not be equated directly with autophagic flux. As emphasized in methodological and conceptual papers on plant autophagy, the number of observable autophagosomes reflects a dynamic balance among their formation, trafficking, fusion with the vacuole, and degradation, and therefore must be interpreted alongside markers of vacuolar degradation and hydrolytic capacity [50,51,60,61]. In this respect, the combined use of ultrastructural observations, MDC staining, transcriptomics, proteomics, Western blotting, and proteolytic assays is a substantial strength of our work, because it reveals that asparagine affects several components of the pathway simultaneously rather than producing a simple on/off effect. Accordingly, in the present study, autophagic flux is inferred indirectly from convergent microscopy, ATG8 immunoblotting, transcriptomic, proteomic, and proteolytic readouts rather than measured with a dedicated flux reporter. This limitation does not weaken the integrated interpretation, but it indicates that future work should include reporter-based flux assays to resolve whether asparagine primarily affects autophagosome formation, trafficking, fusion with the vacuole, or vacuolar degradation.

Such a multi-level effect is physiologically plausible in germinating lupin tissues, where asparagine is a central component of nitrogen storage, transport, and remobilization. Notably, the physiological relevance of this amino acid in the lupin embryonic axis is supported by our earlier measurements, which showed its significant accumulation under starvation conditions, especially in −S and −S+Asn embryonic axes of Andean lupin [22]. This point strengthens the interpretation that the effects observed after asparagine supplementation are superimposed on an endogenous asparagine-based metabolic background rather than representing an entirely artificial response. Under carbon deficit, the embryonic axis must maintain viability while coping with a high nitrogen load released from storage protein breakdown. In this context, asparagine may contribute to balancing nutrient recycling and nitrogen conservation by attenuating selected autophagy- and proteolysis-related responses and favoring the retention of nitrogen in a readily transportable form. This interpretation is consistent with the established role of asparagine metabolism as a marker of high cellular N:C status, particularly under carbon limitation or darkness in heterotrophic plant tissues [55,56,57,58,62]. It is also compatible with broader models in which nutrient signaling pathways, particularly SnRK1- and TOR-related pathways, coordinate carbon and nitrogen status with the control of autophagy induction and autophagic flux [21,23,63,64,65]. In the present experimental system, exogenous asparagine cannot be assigned exclusively to either a metabolic-substrate role or a signaling role. Its effects probably reflect an integrated response to increased nitrogen availability under carbon limitation; discriminating between these two functions would require additional approaches, such as isotope-labeled asparagine tracing, manipulation of asparagine synthetase or asparaginase activity, and direct analysis of nutrient-signaling components.

This interpretation also helps explain why asparagine enhances total antioxidant activity in sugar-starved axes but reduces it in sucrose-fed ones (Figure 2(Ac,Bc)). Under carbon-sufficient conditions, exogenous asparagine likely shifts metabolism away from a starvation-type stress program. Under carbon deficit, by contrast, asparagine appears to be associated with a redox-stabilizing state, as reflected in higher total antioxidant activity and increased catalase content (Figure 2(Ab,Bb)), together with reduced proteolytic activity and lower vacuolar hydrolase-related responses (Table 3; Figure 7). Thus, the antioxidant effect of asparagine should not be interpreted as an isolated response but rather as part of a coordinated metabolic adjustment in which autophagy, proteolysis, and redox homeostasis are rebalanced according to trophic conditions. This view is consistent with current concepts showing that ROS metabolism and autophagy are tightly interconnected, and that both ROS production/scavenging and autophagic activity are modulated by cellular metabolic and energy status, particularly under carbon limitation and other stress conditions [21,23,25,50].

### 3.3. Conserved and Species-Specific Responses in White and Andean Lupin

A major strength of the present study is that this asparagine-guided regulatory pattern was analyzed comparatively in two lupin species. The common features of the response in white lupin (an Old-World lupin species) and Andean lupin (a New-World lupin species) indicate that crosstalk among carbon starvation, redox remodeling, autophagy, and asparagine action is a conserved feature of lupin embryonic axes. In both species, carbon starvation enhanced the autophagy-related response, increased proteolytic activity, and altered ROS/antioxidant components, whereas asparagine under starvation conditions reduced proteolytic activity and improved antioxidant potential. These shared responses strongly suggest that asparagine does not act in a species-specific or incidental manner but rather represents a broader metabolic regulator of the starvation response in germinating lupin tissues [15,22,24]. At the same time, the differences between the two species remain informative and biologically meaningful. White lupin generally showed a clearer asparagine-dependent decrease in transcript levels of *ATG* genes (Figure 3(Aa,Bb)), vacuolar proteases (Figure 5A), hydrolases (Figure 6A), and vacuolar lipases [15] together with a marked increase in GOX protein content (Figure 1(Ab), Table 1). In Andean lupin, starvation more strongly enhanced the transcript abundance of genes encoding ROS-producing proteins (Figure 1(Ba)), while ATG8 protein content decreased under starvation (Figure 3(Bc)), which may be consistent with intensified turnover of this marker protein during active autophagic flux, because ATG8 can be delivered to the vacuole and degraded during autophagy progression [11,49,51]. Thus, the two species appear to share the same basic regulatory framework but differ in the relative intensity with which their redox-related, autophagy-related, and vacuolar hydrolytic components are engaged, consistent with previous evidence that sugar starvation induces autophagy and peroxisome-related remodeling in lupin embryonic axes [22,24]. These interspecific differences are plausible in view of the distinct reserve composition and metabolic characteristics of the two lupins. White lupin seeds are typically rich in protein and comparatively lower in oil, whereas Andean lupin is characterized by both high protein content and distinctly higher lipid accumulation [36,37,38,39,40,41]. Such differences are expected to affect the relative contributions of amino acid remobilization, peroxisomal lipid catabolism, and ROS-producing reactions during post-germinative growth. In this context, the stronger starvation-induced response in transcripts of genes encoding ROS-generating proteins (Figure 1(Ba)) and the changes in acyl-CoA oxidase, an enzyme associated with peroxisomal fatty acid β-oxidation (Table 1), in Andean lupin may reflect greater engagement of lipid-associated and peroxisome-linked metabolism. Conversely, the stronger response of white lupin to asparagine in the transcriptional profile of vacuolar hydrolases may indicate tighter coupling between nitrogen remobilization and vacuolar catabolic control, which is consistent with recent evidence that germination and seedling establishment in white lupin involve extensive proteome reorganization and coordinated amino-acid and reserve metabolism [66]. From an applied perspective, the present findings also support the broader view that adjustment of carbon allocation and C:N balance may influence stress resilience. Recent work showing that optimized carbon partitioning can improve crop heat tolerance without compromising quality further illustrates the translational relevance of mechanisms that coordinate carbon availability, stress responses, and metabolic homeostasis [67]. Although this interpretation remains tentative, it offers a coherent physiological framework for understanding why the same trophic treatments cause both conserved and species-specific effects in lupin embryonic axes [22,24].

## 4. Materials and Methods

### 4.1. Plant Material

The experiments were conducted using in vitro-cultured embryonic axes isolated from germinating seeds of white lupin (*Lupinus albus* L.) and Andean lupin (*Lupinus mutabilis* Sweet). The in vitro culture process was described in detail earlier [22,24]. Briefly, isolated embryonic axes were maintained under sterile conditions for 96 h in the dark at 25 °C, using liquid mineral Heller medium [68] containing (+S) or lacking (−S) 60 mM sucrose, with (+Asn) or without 35 mM asparagine. The 60 mM sucrose and 35 mM asparagine concentrations, and the 96 h sampling point were selected based on our previous studies with isolated lupin embryonic axes, in which these conditions produced reproducible physiological and cytological responses under sugar-starvation conditions and allowed direct comparison with earlier experiments. The morphology and detailed physio-morphological parameters of the embryonic axes have been reported previously [22,24]. In experiments with the autophagy inhibitor concanamycin A (ConA), after 72 h of in vitro culture, embryonic axes were transferred to Eppendorf tubes containing the appropriate medium supplemented with 10 μM ConA dissolved in 0.1 mM DMSO, as in the previously established protocol, and cultured under the same external conditions for a further 24 h [24].

### 4.2. Transcriptomics–NGS and qRT-PCR

#### 4.2.1. NGS (Next-Generation Sequencing)

NGS-based quantitative transcriptomic analysis was performed following the detailed procedure described by Borek et al. [24]. In brief, 96 h in vitro-cultured lupin embryonic axes were frozen in liquid nitrogen and stored at −80 °C until RNA isolation. Total RNA was isolated from powdered, frozen embryonic axes (50 mg) using the RNeasy Plant Mini Kit (Qiagen, Hilden, Germany). After assessment of RNA quality and quantity, equal amounts (4 µg) of total RNA from each sample were used to prepare cDNA libraries. NGS cDNA libraries were prepared from purified mRNA using the TruSeq Stranded mRNA Sample Preparation kit v2 (RS-122-2101, Illumina, San Diego, CA, USA) according to the manufacturer’s protocol. Before high-throughput NGS, library quality and pooling were assessed on the MiSeq (preliminary run using 50 bp). Libraries were sequenced using a 150 bp paired-end protocol. The in vitro culture of the embryonic axes, RNA isolation, and NGS were performed with three independent replicates. Raw transcriptomic data have been deposited in the SRA database (accession numbers: PRJNA953600 for white lupin https://www.ncbi.nlm.nih.gov/sra/PRJNA953600 (accessed on 11 April 2023) and PRJNA953433 for Andean lupin https://www.ncbi.nlm.nih.gov/sra/PRJNA953433 (accessed on 9 April 2023)). After demultiplexing, raw sequencing data were cleaned by eliminating adapter reads, N-base reads, and low-quality reads using the CLC Genomics Workbench trim sequences module (Qiagen version 20). The expression level of each gene in each library was calculated by quantifying the number of Illumina reads that mapped to the LupAngTanjil_v1.0 reference sequence annotated with cDNAs (57,263 contigs) using the CLC Genomics Workbench RNA-Seq Analysis module. The raw gene expression counts were normalized using the RPKM (reads per kilobase of the exon model per million mapped reads) method described by Mortazavi et al. [69]. Heatmaps for selected records were generated using the online software Heatmapper (https://server.heatmapper2.ca (accessed on 14 April 2026)), based on normalized means from three independent sequencing runs (Supplementary Tables S1–S3, S5, and S7).

#### 4.2.2. qRT-PCR

Total RNA was isolated from 200 mg of frozen embryonic axes (−80 °C). Samples were ground in liquid nitrogen using the RNeasy Plant Mini Kit (Qiagen) according to the manufacturer’s instructions. RNA concentration and purity were assessed with a NanoDrop 2000 spectrophotometer (Thermo Scientific, Waltham, MA, USA), and only samples with A260/280 ratios of 1.9–2.1 were used. RNA integrity was verified by agarose gel electrophoresis, and purity was confirmed by PCR with actin-specific primers (Supplementary Table S8). For cDNA synthesis, 3 μg of total RNA was reverse-transcribed using oligo(dT)20 primers and SuperScript III Reverse Transcriptase (Invitrogen). qRT-PCR was performed on a CFX96 Real-Time PCR Detection System (Bio-Rad) using iTaq Universal SYBR Green Supermix (Bio-Rad) and gene-specific primers (Supplementary Table S8) designed from NGS-derived sequences mapped to the reference genome. Relative gene expression was calculated using the 2^−ΔΔCT^ method [70] with actin as the reference gene, and results were expressed relative to the +S trophic variant calibrator. Actin was selected after verification of stable expression under the experimental conditions based on NGS-derived expression data across the analyzed trophic variants and preliminary PCR assessment.

### 4.3. Proteomics–iTRAQ, Western Blot, and Proteolytic Activity

#### 4.3.1. iTRAQ (Isobaric Tags for Relative and Absolute Quantitation)

iTRAQ-based quantitative proteomic analysis was performed at the Mass Spectrometry Laboratory at the Institute of Biochemistry and Biophysics of the Polish Academy of Sciences in Warsaw, following the detailed procedure described by Borek et al. [24]. In brief, lupin embryonic axes were homogenized, and protein was precipitated in 10% trichloroacetic acid (TCA) in acetone at −20 °C. 100 µg of protein per sample was prepared using the FASP protocol with minor adjustments [71]. Next, the proper amount of peptides resuspended in 30 µL of 100 mM TEAB was labeled with iTRAQ 8-plex (SCIEX, Framingham, MA, USA). iTRAQ-labeled peptides were fractionated using high-pH reverse-phase chromatography on an XBridge Peptide BEH C18 column (4.6 × 250 mm, 130 Å, 5 µm, Waters Corporation, Milford, MA, USA). Twenty-five fractions were analyzed using an LC-MS system composed of a UPLC chromatograph (nanoAcquity, Waters Corporation, Milford, MA, USA) coupled to a Q Exactive mass spectrometer (Thermo Scientific, Waltham, MA, USA). The mass spectrometry proteomics data have been deposited to the ProteomeXchange Consortium via the PRIDE [46] partner repository with the dataset identifier PXD041380, Project DOI: 10.6019/PXD041380, https://www.ebi.ac.uk/pride/archive/projects/PXD041380 (accessed on 6 April 2023). The acquired MS/MS data were pre-processed using Mascot Distiller (v. 2.6, Matrix Science, London, UK), and database searches were performed using the Mascot Search Engine (Matrix Science, London, UK, Mascot Server 2.5) against the NCBInr Lupinus database (61,158 sequences; 33,890,079 residues). Statistical significance was determined using the in-house software package Diffprot [45]. Only proteins with a q-value below 0.05 or proteins present in only one of the two comparative groups were included in subsequent analyses.

#### 4.3.2. Western Blot

Immunoquantification of ATG8, catalase (Cat), and glycolate oxidase (GOX) was performed as described by Borek et al. [24]. Embryonic axes frozen in liquid nitrogen and stored at −80 °C were homogenized on ice in extraction buffer (7 M urea, 2 M thiourea, 4% CHAPS, 35 mM Tris) at a 1:2 (*w*/*v*) ratio. Protein content was determined using the Bradford method [72], with bovine serum albumin (BSA) as the standard. Samples containing 15 (for ATG8) or 40 (for Cat and GOX) µg of total protein were denatured in Laemmli buffer with 25 mM DTT at 85 °C for 8 min or 90 °C for 3 min and were separated on a 12.5–18% SDS-PAGE gel [73] using the Mini-PROTEAN Tetra Cell (Bio-Rad Laboratories, Inc., Grand Junction, CO, USA). Separated proteins were transferred to a PVDF membrane (Immobilon-P, pore size of 0.45 µm) at a constant 1.25–2.5 A for 10 min using a Trans-Blot Turbo transfer system (Bio-Rad Laboratories, Inc., Grand Junction, CO, USA). Equal sample loading and even transfer were controlled with Ponceau S staining. Blots were blocked in 5% skim milk or 3% BSA in Tris-buffered saline (TBS) for 2 h at room temperature or overnight at 4 °C with agitation. Subsequently, blots were incubated with the primary antibody Anti-ATG8, Anti-Cat, or Anti-GOX (Agrisera, Vännäs, Sweden, catalog no. AS14 2769, AS09 501, and AS14 2772, respectively) at a dilution of 1:1000–1:10,000 in a blocking solution containing 1% skim milk or 1% BSA in TBS, for 2 h at room temperature or overnight at 4 °C with agitation. After removal of the primary antibody solution, the blot was washed three times for 5 min in TBS at room temperature with agitation. The membrane was then incubated with the secondary antibody solution (goat anti-rabbit IgG horseradish peroxidase (HRP) conjugated, Agrisera, Vännäs, Sweden) at a dilution of 1:10,000 in a blocking solution containing 1% skim milk or 1% BSA in TBS for 2 h at room temperature with agitation. After incubation, the blot was washed three times for 5 min in TBS at room temperature. Bands were imaged digitally using the ChemiDoc MP Imaging System (Bio-Rad, Hercules, CA, USA) after incubation for 120–180 s with ECL SuperBright (Agrisera, Vännäs, Sweden). Exposure times were 20–60 s. Representative full membranes are provided in the Supplementary Figure S1.

#### 4.3.3. Proteolytic Activity

Proteolytic activity was measured colorimetrically at 405 nm by monitoring nitroanilide (NA) released from substrates specific for exopeptidases (L-alanine-p-nitroanilide hydrochloride, Ala-p-NA, and L-leucine-p-nitroanilide, Leu-NA) and endopeptidases (N-acetyl-L-alanine-p-nitroanilide, A-Ala-p-NA, and N-acetyl-L-leucine-p-nitroanilide, A-Leu-p-NA). Embryonic axes were homogenized in Tris-HCl buffer (100 mM, pH 7.0, 0.1% β-mercaptoethanol) at a 1:3 (*w*/*v*) ratio and centrifuged at 22,000 g. Substrate solutions were prepared fresh (4 mg dissolved in 1 mL of 100% methanol, and 1 mL of homogenization buffer added), and the incubation mixture (2.5 mL) contained: homogenization buffer, 400 μL of substrate solution, and 10–400 μL of the supernatant. Kinetic reactions were monitored using the spectrophotometer Ultrospec 4000 (Pharmacia Biotech, Cambridge, UK) for 5 and 15 min for the exopeptidase and endopeptidase activity assays, respectively. Protein content in enzyme extracts was determined using the Bradford method [72], with bovine serum albumin (BSA) as the standard.

### 4.4. Antioxidant Activity

Antioxidant activity was measured according to the method of Brand-Williams et al. [74]. Lupin embryonic axes were homogenized at a 1:3 (*w*/*v*) ratio in 80% methanol, incubated for 2 h at 37 °C, and centrifuged at 12,000 g. The reaction mixtures consisted of 100 µL of supernatant, 1000 µL of 80% methanol, and 500 µL of 0.4 mM 2,2-diphenyl-1-picrylhydrazyl (DPPH), and absorbance was measured using the spectrophotometer Ultrospec 4000 (Pharmacia Biotech) at 517 nm. Subsequently, the samples were incubated for 20 min at room temperature in the dark, and absorbance was measured again at the same wavelength. Antioxidant activity was expressed as the percentage difference in DPPH quenching between the initial and final absorbance readings.

### 4.5. Confocal Microscopy

Autophagosomes were stained with monodansylcadaverine (MDC) as described by Pu and Bassham [60]. Approximately 3 mm long root tips of embryonic axes were incubated in 0.2 μM MDC dissolved in PBS buffer for 10 min in the dark. Observations were performed using a confocal microscope, LSM 510 (Carl Zeiss Microscopy GmbH, Jena, Germany), with excitation at 335 nm and emission at 505–550 nm.

### 4.6. Statistical Analysis

Results shown in the graphs are means ± SD from three independent biological replicates. Data were subjected to one-way ANOVA followed by Tukey’s HSD multiple-range test using Statistica software Version 13 (TIBCO Software Inc., Palo Alto, CA, USA). iTRAQ and RNA-Seq (NGS) were performed in three independent biological replicates. Statistical analysis of iTRAQ data was performed using Diffprot software [45], which implements a non-parametric test for significance with correction for multiple testing.

## 5. Conclusions

The present study indicates that sugar starvation activates a survival-oriented catabolic program in lupin embryonic axes, involving redox remodeling, autophagy, vacuolar proteolysis, and changes in hydrolytic capacity. Within this program, asparagine emerges as an important metabolic regulator closely linked to the characteristic nitrogen status of germinating lupin seeds. Rather than indicating an effect restricted to a single autophagic step, the present data suggest that asparagine is associated with coordinated changes in several autophagy-related parameters, including delayed vacuolar degradation of autophagic bodies, reduced numbers of detectable autophagosomes, lower vacuolar protease- and hydrolase-related responses, and enhanced antioxidant capacity under carbon deficit. Thus, asparagine may help sugar-starved embryonic axes maintain metabolic homeostasis by coordinating nitrogen preservation, redox stabilization, and controlled intracellular recycling.

The comparison of white and Andean lupin further shows that this regulatory pattern is not species-specific, although the two species differ in the relative intensity of individual components of the response. In both species, sugar starvation promoted autophagy-related and proteolytic responses and affected redox-associated pathways, whereas asparagine reduced proteolytic capacity and modified autophagy-related parameters under starvation. At the same time, Andean lupin showed a stronger starvation-induced transcriptional response of ROS-generating proteins, while white lupin displayed a clearer asparagine-dependent transcriptional response of vacuolar hydrolases. These differences probably reflect distinct reserve composition and metabolic strategies of the two species, including different contributions of lipid-associated, peroxisome-linked, and nitrogen-remobilization pathways during post-germinative growth.

Because only one exogenous asparagine concentration and one 96 h sampling point were used, the present study does not define a dose–response relationship or the temporal sequence of asparagine-dependent responses during imbibition, radicle protrusion, and early seedling development. Future work should address which component of the asparagine-dependent response is primary. In particular, it will be important to determine whether asparagine directly affects vacuolar function, vacuolar pH, V-ATPase activity, and the activity of vacuolar proteases, lipases, and other hydrolases. It will also be necessary to clarify whether the lower number of detectable autophagosomes under asparagine treatment reflects altered autophagosome formation, trafficking, fusion with the vacuole, or faster/modified turnover. Additional autophagic flux markers, together with analyses of ATG8 processing and vacuolar degradation, would help distinguish among these possibilities. Another important direction will be a more detailed analysis of asparagine metabolism itself, particularly its compartmentation, turnover, and relationship to asparagine synthetase and asparaginase activities. Finally, the potential involvement of nutrient-signaling modules, such as SnRK1- and TOR-related pathways, should be examined to define more precisely how carbon starvation and nitrogen-rich metabolites are integrated during early seedling establishment in lupins.

## Figures and Tables

**Table 1:** Relations among ROS-generating protein contents determined by the iTRAQ method in embryonic axes isolated from germinating seeds of white and Andean lupin and cultured in vitro for 96 h on a medium with (+S) or without (−S) 60 mM sucrose. Culture media were also enriched with 35 mM asparagine (+Asn). Protein quantification data were compared across the following pairs of axes: +S/+S+Asn, +S/−S, and −S/−S+Asn.

+S/+S+Asn				+S/−S				−S/−S+Asn				Protein ID/NCBI	Description
q-Value	Ratio A/B	Fold Change	Peptide Number	q-Value	Ratio A/B	Fold Change	Peptide Number	q-Value	Ratio A/B	Fold Change	Peptide Number		
White lupin													
0.78650	1.12	1.12	7	0.01655	1.43	1.43	6	0.18367	1.36	1.36	5	XP_019424711.1	probable NAD(P)H dehydrogenase (quinone) FQR1-like 1
0.78706	1.06	1.06	12	0.00847	0.8	1.24	16	0.63894	1.22	1.22	12	XP_019449047.1	acyl-coenzyme A oxidase 3, peroxisomal
0.94229	1.06	1.06	6	0.00006	0.55	1.82	11	0.60044	1.08	1.08	12	XP_019446061.1	acyl-coenzyme A oxidase 4, peroxisomal-like
1.00000	0.96	1.04	10	0.00783	0.73	1.37	12	0.95420	1.07	1.07	10	XP_019446158.1	peroxisomal (S)-2-hydroxy-acid oxidase GLO1-like (GOX)
1.00000	1.08	1.08	2	0.02564	0.69	1.46	5	1.00000	1.12	1.12	2	XP_019443786.1	peroxisomal (S)-2-hydroxy-acid oxidase GLO1-like (GOX)
0.95292	0.99	1.01	7	0.00197	0.7	1.44	9	1.00000	0.98	1.02	10	XP_019440288.1	quinone oxidoreductase PIG3-like
Andean lupin													
1.00000	1.05	1.05	5	0.00041	1.95	1.95	6	0.57452	0.85	1.17	5	XP_019419082.1	probable NAD(P)H dehydrogenase (quinone) FQR1-like 1
0.32737	1.07	1.07	12	0.01996	0.82	1.22	14	0.19690	1.21	1.21	14	XP_0194490471	acyl-coenzyme A oxidase 3, peroxisomal
1.00000	0.97	1.03	2	0.03177	1.71	1.71	4	0.93652	0.99	1.01	2	XP_019458479.1	peroxidase 3-like
1.00000	0.95	1.06	13	0.04838	1.18	1.18	16	0.00037	0.67	1.48	17	XP_019452293.1	NADH-cytochrome b5 reductase-like protein

Statistical analysis was performed using Diffprot software [45], which implements a nonparametric test to assess significance with a correction for multiple testing. The differences in protein levels are statistically significant when the q-value is below 0.05. The pink filling of the table cells indicates a statistically significant increase, and the blue filling of a cell indicates a statistically significant decrease (in the B component of the A/B ratio) in the protein level in axes cultured in vitro under different nutrient conditions. The grey filling of a cell indicates changes that did not reach statistical significance. The data represent results that were obtained from three independent experiments. The full iTRAQ data were presented in Supplementary Table S1 in Borek et al. 2023, and the raw mass spectrometry proteomics data for white and Andean lupin embryonic axes have been deposited to the ProteomeXchange Consortium via the PRIDE [46] partner repository with the dataset identifier PXD041380. Project doi:10.6019/PXD041380, https://www.ebi.ac.uk/pride/, deposited on 6 April 2023.

**Table 2:** Relations among antioxidant protein levels determined by the iTRAQ method in embryonic axes isolated from germinating seeds of white and Andean lupin and cultured in vitro for 96 h on a medium with (+S) or without (−S) 60 mM sucrose. Culture media were also enriched with 35 mM asparagine (+Asn). Protein quantification data were compared across the following pairs of axes: +S/+S+Asn, +S/−S, and −S/−S+Asn.

+S/+S+Asn				+S/−S				−S/−S+Asn				Protein ID/NCBI	Description
q-Value	Ratio A/B	Fold Change	Peptide Number	q-Value	Ratio A/B	Fold Change	Peptide Number	q-Value	Ratio A/B	Fold Change	Peptide Number		
White lupin													
0.37215	1.1	1.1	5	0.15639	0.82	1.22	6	1.00000	1.09	1.09	6	XP_019453379.1	catalase isozyme 1-like
1.00000	0.95	1.05	12	0.04037	0.85	1.18	17	0.09577	0.83	1.21	13	XP_019445665.1	monothiol glutaredoxin-S17
1.00000	0.87	1.15	5	0.02933	1.34	1.34	5	1.00000	0.9	1.12	5	XP_019454014.1	L-ascorbate peroxidase, cytosolic
1.00000	1.02	1.02	4	0.0077	0.56	1.78	4	0.25649	0.82	1.22	5	XP_019420237.1	probable phospholipid hydroperoxide glutathione peroxidase
0.08531	0.83	1.2	3	0.0003	0.39	2.56	5	0.0446	0.74	1.35	6	XP_019463244.1	1-Cys peroxiredoxin
1.00000	0.95	1.06	4	0.00822	0.82	1.23	14	1.00000	1.32	1.32	12	XP_019444263.1	2-Cys peroxiredoxin BAS1, chloroplastic-like
												XP_019443004.1	2-Cys peroxiredoxin BAS1, chloroplastic
0.00505	1.11	1.11	18	0.00006	1.47	1.47	16	1.00000	0.95	1.05	15	XP_019429896.1	monodehydroascorbate reductase 5, mitochondrial
0.47107	0.95	1.05	4	0.04503	1.5	1.5	4	1.00000	1.22	1.22	4	XP_019423334.1	glutathione S-transferase U17-like
1.00000	0.95	1.06	4	0.01424	0.7	1.42	6	1.00000	1.15	1.15	7	XP_019436148.1	glutathione S-transferase zeta class-like isoform X1
												XP_019436150.1	glutathione S-transferase zeta class-like isoform X2
0.83983	0.91	1.1	5	0.00172	0.56	1.77	6	1.00000	1.05	1.05	2	XP_019443778.1	probable glutathione S-transferase
0.58151	0.89	1.12	2	0.04288	0.61	1.65	3	1.00000	0.99	1.01	2	XP_019459310.1	probable glutathione S-transferase
1.00000	0.98	1.02	2	0.00135	0.27	3.76	3	1.00000	0.88	1.14	6	XP_019418295.1	probable glutathione S-transferase parA
1.00000	0.95	1.06	3	0.03045	0.61	1.63	3	1.00000	1.3	1.3	3	XP_019432363.1	probable glutathione S-transferase parC
0.02796	0.89	1.12	6	0.42091	0.86	1.17	5	0.95291	0.97	1.03	5	XP_019423645.1	thioredoxin H-type-like
Andean lupin													
1.00000	1.09	1.09	7	0.49806	0.9	1.11	4	0.00149	0.47	2.12	7	XP_019453379.1	catalase isozyme 1-like
1.00000	1.09	1.09	12	0.00456	1.32	1.32	13	0.39864	0.91	1.1	7	XP_019438583.1	probable L-ascorbate peroxidase 6, chloroplastic
1.00000	0.97	1.03	2	0.03177	1.71	1.71	4	0.93652	0.99	1.01	2	XP_019458479.1	peroxidase 3-like
1.00000	0.81	1.23	2	1.00000	0.75	1.33	3	0.00596	0.45	2.24	4	XP_019443533.1	peroxiredoxin Q, chloroplastic-like
1.00000	1.08	1.08	12	0.00008	1.92	1.92	9	0.04342	1.35	1.35	11	XP_019429896.1	monodehydroascorbate reductase 5, mitochondrial
1.00000	1.05	1.05	13	1.00000	1.05	1.05	11	0.00110	1.54	1.54	13	XP_019444265.1	monodehydroascorbate reductase, seedling isozyme-like
1.00000	1.01	1.01	7	0.01755	1.32	1.32	14	0.49218	0.64	1.55	2	XP_019422106.1	thioredoxin reductase NTRB-like
												XP_019424108.1	thioredoxin reductase NTRB-like
1.00000	1.2	1.2	4	1.00000	1.15	1.15	3	0.01728	1.82	1.82	4	XP_019450278.1	probable glutathione S-transferase

Statistical analysis was performed using Diffprot software [45], which implements a nonparametric test to assess significance with a correction for multiple testing. The differences in protein levels are statistically significant when the q-value is below 0.05. The pink filling of the table cells indicates a statistically significant increase, and the blue filling of a cell indicates a statistically significant decrease (in the B component of the A/B ratio) in the protein level in axes cultured in vitro under different nutrient conditions. The grey filling of a cell indicates changes that did not reach statistical significance. The data represent results that were obtained from three independent experiments. The full iTRAQ data were presented in Supplementary Table S1 in Borek et al. 2023, and the raw mass spectrometry proteomics data for white and Andean lupin embryonic axes have been deposited to the ProteomeXchange Consortium via the PRIDE [46] partner repository with the dataset identifier PXD041380. Project doi:10.6019/PXD041380, https://www.ebi.ac.uk/pride/, deposited on 6 April 2023.

**Table 3:** Relations in hydrolase contents determined by the iTRAQ method in embryonic axes isolated from germinating seeds of white and Andean lupin and cultured in vitro for 96 h on a medium with (+S) or without (−S) 60 mM sucrose. Culture media were also enriched with 35 mM asparagine (+Asn). Protein quantification data were compared across the following pairs of axes: +S/+S+Asn, +S/−S, and −S/−S+Asn. DeepLoc 2.0 software (https://services.healthtech.dtu.dk/services/DeepLoc-2.0/ (accessed on 11 March 2025)), which uses a high-quality model, was used (Supplementary Tables S4 and S6).

+S/+S+Asn				+S/−S				−S/−S+Asn				Protein ID/NCBI	Description and Predicted Localization
q-Value	Ratio A/B	Fold Change	Peptide Number	q-Value	Ratio A/B	Fold Change	Peptide Number	q-Value	Ratio A/B	Fold Change	Peptide Number		
White lupin													
0.47795	1.04	1.04	14	0.00006	0.7	1.42	21	0.36	1.16	1.16	17	XP_019430110.1	probable alpha-mannosidase At5g13980 (vacuole)
1.00000	1.04	1.04	12	0.00012	0.73	1.37	15	1.00	1.00	1.00	16	XP_019447409.1	probable alpha-mannosidase At5g13980 (vacuole)
1.00000	0.96	1.04	11	0.00072	1.43	1.43	11	0.04	0.81	1.24	9	XP_019440165.1	glucan endo-1.3-beta-glucosidase, basic isoform-like (extracellular)
1.00000	0.99	1.01	6	0.04314	1.27	1.27	9	1.00	1.17	1.17	3	XP_019456072.1	beta-glucosidase 42 (cytoplasm)
1.00000	1.04	1.04	11	0.01578	1.32	1.32	7	1.00	1.10	1.10	8	XP_019448091.1	phospholipase D alpha 1-like (cytoplasm, cell membrane, vacuole)
0.97535	1.08	1.08	9	0.14173	1.17	1.17	10	0.04	1.22	1.22	9	XP_019435653.1	glycerophosphodiester phosphodiesterase GDPDL3-like (cell membrane)
1.00000	0.99	1.01	5	0.01397	0.7	1.43	7	1.00	0.75	1.33	7	XP_019416817.1	endochitinase A2 (extracellular)
1.00000	1	1	6	0.01551	0.68	1.47	9	1.00	1.06	1.06	9	XP_019441211.1	chitinase-a (extracellular)
0.97535	1.03	1.03	3	0.01938	0.68	1.46	5	0.90685	0.85	1.17	5	XP_019422329.1	proline carboxypeptidase (lysosomal Pro-X carboxypeptidase) isoform X1 (vacuole)
												XP_019422330.1	proline carboxypeptidase (lysosomal Pro-X carboxypeptidase) isoform X2 (vacuole)
0.00043	1.29	1.29	9	0.01119	0.67	1.5	8	0.00189	1.73	1.73	8	XP_019446808.1	cysteine proteinase COT44-like (vacuole)
1.00000	1.01	1.01	7	0.01265	1.42	1.42	8	0.65378	0.87	1.15	4	XP_019412989.1	subtilisin-like protease SBT1.6 (extracellular)
0.70698	1.04	1.07	10	0.01505	1.28	1.28	12	1.00000	0.99	1.01	7	XP_019461072.1	subtilisin-like protease SBT1.5 (vacuole)
0.99906	1.09	1.09	2	0.04956	1.69	1.69	3	0.85247	0.90	1.11	2	XP_019454630.1	subtilisin-like protease SBT2.5 (extracellular, vacuole)
Andean lupin													
1.00000	1.3	1.3	7	0.00129	1.57	1.57	7	0.76	0.98	1.02	7	XP_019440727.1	bifunctional purple acid phosphatase 26 isoform X1 (extracellular)
												XP_019440736.1	bifunctional purple acid phosphatase 26 isoform X2 (extracellular, vacuole)
												XP_019440745	bifunctional purple acid phosphatase 26 isoform X3 (extracellular, vacuole)
												XP_019440753.1	bifunctional purple acid phosphatase 26 isoform X4 (extracellular, vacuole)
1.00000	0.97	1.03	8	0.82985	0.70	1.43	9	0.00374	0.66	1.51	10	XP_019416817.1	endochitinase A2 (extracellular)
1.00000	1.16	1.16	10	0.82756	0.98	1.02	18	0.01569	1.36	1.36	13	XP_019443381.1	phospholipase D alpha 1 (cytoplasm, cell membrane, vacuole)
1.00000	0.95	0.95	7	0.28550	0.83	1.20	7	0.01882	0.72	1.39	7	XP_019419019.1	cathepsin B-like (vacuole)
1.00000	1.07	1.07	7	0.20056	1.27	1.27	7	0.04096	0.78	1.28	9	XP_019427925.1	subtilisin-like protease SBT1.4 (endoplasmic reticulum)
1.00000	1.04	1.04	4	0.04706	1.54	1.54	5	0.86032	1.02	1.02	6	XP_019431846.1	subtilisin-like protease SBT1.7 (extracellular)

Statistical analysis was performed using Diffprot software [45], which implements a nonparametric test to assess significance with a correction for multiple testing. The differences in protein levels are statistically significant when the q-value is below 0.05. The pink filling of the table cells indicates a statistically significant increase, and the blue filling of a cell indicates a statistically significant decrease (in the B component of the A/B ratio) in the protein level in axes cultured in vitro under different nutrient conditions. The grey filling of a cell indicates changes that did not reach statistical significance. The data represent results that were obtained from three independent experiments. The full iTRAQ data were presented in Supplementary Table S1 in Borek et al. 2023, and the raw mass spectrometry proteomics data for white and Andean lupin embryonic axes have been deposited to the ProteomeXchange Consortium via the PRIDE [46] partner repository with the dataset identifier PXD041380. Project doi:10.6019/PXD041380, https://www.ebi.ac.uk/pride/, deposited on 6 April 2023.

## Data Availability

The mass spectrometry proteomics data for white and Andean lupin embryonic axes have been deposited to the ProteomeXchange Consortium via the PRIDE [46] partner repository with the dataset identifier PXD041380, Project doi:10.6019/PXD041380, https://www.ebi.ac.uk/pride/, deposited on 6 April 2023. Transcriptomics data (NGS) have been deposited to the SRA database. The BioProject accession number for white lupin: PRJNA953600, https://www.ncbi.nlm.nih.gov/sra/PRJNA953600, deposited on 11 April 2023, and Andean lupin: PRJNA953433, https://www.ncbi.nlm.nih.gov/sra/PRJNA953433, deposited on 9 April 2023.

## Supplementary Materials

The following supporting information can be downloaded at: https://www.mdpi.com/article/10.3390/ijms27156851/s1.
