# Supplementary material for: Asparagine-Guided Regulation of Redox Status and Autophagy in Sugar-Starved Lupin (Lupinus spp.) Embryonic Axes—A Transcriptomic and Proteomic Approach
Source: Int J Mol Sci. 2026 Jul 30;27(15):6851. doi: 10.3390/ijms27156851 (PMC13467101; doi:10.3390/ijms27156851)
Supplement: Supplementary file 1 [file ijms-27-06851-s001.zip › Supplementary Figure S1 - Western blots, whole membranes.pdf]

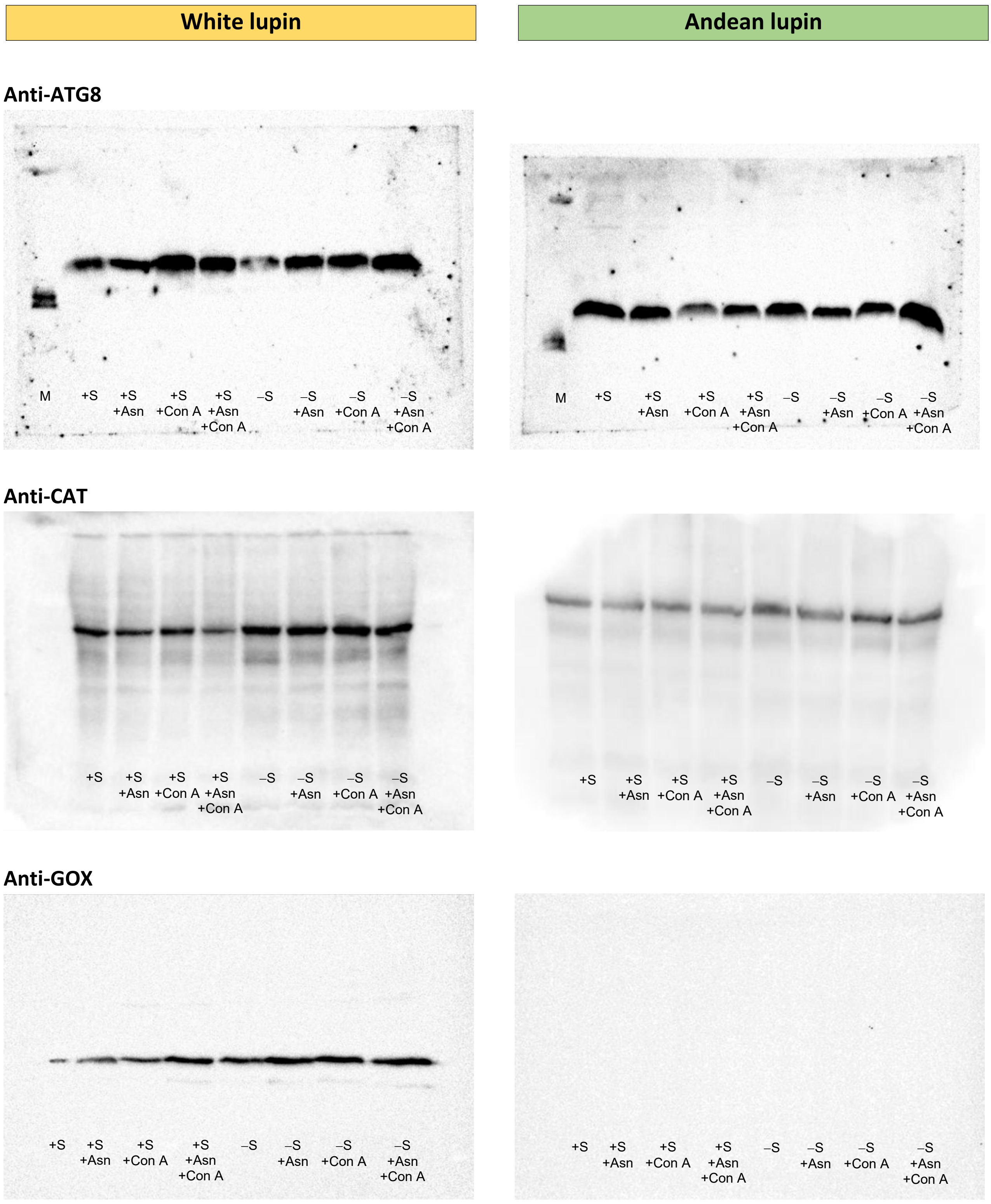

Supplementary Figure S1. Whole membranes from representative Western blots probed with anti-ATG8, anti-CAT, and anti-GOX antibodies. No immunopositive signal was detected with anti-GOX in isolated axes of Andean lupin. Embryonic axes were isolated from germinating seeds and kept under sterile conditions for 96 hours in the dark at 25°C in a mineral liquid medium with (+S) or without (-S) 60 mM sucrose and with (+Asn) or without 35 mM asparagine. In Andean lupin axes, no positive signal was obtained using anti-GOX antibodies. CAT, catalase; GOX, glycolate oxidase.
