# Supplementary material for: Asparagine-Guided Regulation of Redox Status and Autophagy in Sugar-Starved Lupin (Lupinus spp.) Embryonic Axes—A Transcriptomic and Proteomic Approach
Source: Int J Mol Sci. 2026 Jul 30;27(15):6851. doi: 10.3390/ijms27156851 (PMC13467101; doi:10.3390/ijms27156851)
Supplement: Supplementary file 1 [file ijms-27-06851-s001.zip › Supplementary Table S4 - Subcellular localization - proteases.pdf]

Supplementary Table S4. Prediction of subcellular localization of **proteases** in white and Andean lupin embryonic axes. DeepLoc 2.0 software (https://services.healthtech.dtu.dk/services/DeepLoc-2.0/) with a high-quality model was used. A localization is predicted if its probability is above the threshold value - green filling of a cell. Blue cell filling indicates the best result, but it is below the threshold.

Gene ID	Name	Cytoplasm	Nucleus	Extra-cellular	Cell membrane	Mitochon-drion	Plastid	Endoplasmic reticulum	**Vacuole**	Golgi apparatus	Peroxisome	Lupin species
	**Probability threshold**	**0.4761**	**0.5014**	**0.6173**	**0.5646**	**0.6220**	**0.6395**	**0.6090**	**0.5848**	**0.6494**	**0.7364**	
XM_019586494.1	carboxypeptidase SOL1-like (LOC109346745)	0.1726	0.1415	0.1084	0.3899	0.0590	0.1918	0.5829	0.5551	0.5871	0.2183	white and Andean
XM_019571716.1	carboxypeptidase SOL1-like (LOC109335576)	0.1666	0.1229	0.1230	0.4122	0.0422	0.0696	0.4909	0.5749	0.5671	0.1510	white and Andean
XM_019572924.1	serine carboxypeptidase-like (LOC109336370)	0.1244	0.0884	0.3709	0.2489	0.0253	0.4313	0.7488	0.7144	0.4975	0.0751	white and Andean
XM_019557206.1	serine carboxypeptidase-like (LOC109325066)	0.1107	0.1299	0.4522	0.2615	0.0206	0.7170	0.4934	0.7045	0.2481	0.0927	white
XM_019592273.1	serine carboxypeptidase-like (LOC109350961)	0.1229	0.1238	0.3534	0.2278	0.0394	0.4099	0.5660	0.8044	0.5941	0.0438	white
XM_019594273.1	serine carboxypeptidase-like (LOC109352335)	0.1368	0.1068	0.4746	0.2187	0.0359	0.5290	0.6533	0.7696	0.4404	0.1916	white
XM_019562414.1	serine carboxypeptidase-like 2 (LOC109328818)	0.1248	0.0757	0.5782	0.2509	0.0092	0.0354	0.5017	0.6331	0.1897	0.0365	white and Andean
XM_019584125.1	serine carboxypeptidase-like 2 (LOC109345246)	0.1063	0.0904	0.3992	0.2142	0.0151	0.0617	0.4915	0.6642	0.2061	0.0310	white and Andean
XM_019598702.1	serine carboxypeptidase-like 7 (LOC109355510)	0.1299	0.0976	0.5621	0.2641	0.0220	0.0546	0.5329	0.6800	0.1763	0.0041	white and Andean
XM_019582531.1	serine carboxypeptidase-like 11 (LOC109343980)	0.1274	0.0889	0.5791	0.2291	0.0139	0.0825	0.5173	0.6944	0.1828	0.0315	white
XM_019579150.1	serine carboxypeptidase-like 13 (LOC109341282)	0.1045	0.0748	0.5583	0.2992	0.0130	0.0622	0.4416	0.5420	0.2058	0.0200	white
XM_019594929.1	serine carboxypeptidase-like 20 (LOC109352738)	0.1235	0.0927	0.4731	0.2303	0.0130	0.0777	0.5445	0.8241	0.2524	0.0226	white
XM_019584333.1	serine carboxypeptidase-like 20 (LOC109345370)	0.1610	0.2104	0.3366	0.1878	0.0197	0.2357	0.6609	0.7181	0.2181	0.0228	white and Andean
XM_019593765.1	serine carboxypeptidase-like 20 (LOC109352003)	0.1094	0.1212	0.5425	0.2902	0.0137	0.1923	0.4570	0.7468	0.2719	0.0405	Andean
XM_019604635.1	serine carboxypeptidase-like 20 (LOC109359944)	0.0956	0.1061	0.5428	0.2384	0.0154	0.1407	0.4215	0.7398	0.1891	0.0247	white and Andean
XM_019558663.1	serine carboxypeptidase 24 (LOC109326030)	0.1230	0.1254	0.4776	0.2165	0.0145	0.1595	0.4599	0.7203	0.2035	0.0240	white and Andean
XM_019574044.1	serine carboxypeptidase-like 25 (LOC109337136)	0.1010	0.1280	0.6724	0.3099	0.0207	0.0670	0.3627	0.7442	0.2897	0.0397	white
XM_019571396.1	serine carboxypeptidase-like 25 (LOC109335266)	0.2108	0.4513	0.6337	0.3461	0.0458	0.2463	0.2022	0.4429	0.1178	0.3313	white
XM_019596474.1	serine carboxypeptidase-like 26 (LOC109354121)	0.5770	0.3645	0.3187	0.0716	0.4497	0.6433	0.1465	0.0184	0.0168	0.2049	white
XM_019586398.1	serine carboxypeptidase-like 27 (LOC109346695)	0.1077	0.0674	0.6104	0.3070	0.0130	0.0472	0.4385	0.7520	0.2910	0.0128	white and Andean
XM_019590095.1	serine carboxypeptidase-like 31 (LOC109349331)	0.1117	0.0619	0.4767	0.2575	0.0188	0.2666	0.4378	0.7998	0.3279	0.0360	white and Andean
XM_019564760.1	serine carboxypeptidase-like 31 (LOC109330538)	0.1003	0.0721	0.4553	0.2767	0.0155	0.2646	0.3676	0.7713	0.2495	0.0505	white
XM_019607728.1	serine carboxypeptidase-like 33 (LOC109362135)	0.1285	0.1470	0.5247	0.2854	0.0191	0.0908	0.4553	0.7619	0.2745	0.0335	white and Andean
XM_019595474.1	serine carboxypeptidase-like 34 (LOC109353271)	0.1052	0.0933	0.5276	0.2931	0.0200	0.1532	0.3947	0.7371	0.2340	0.0286	white and Andean
XM_019557815.1	serine carboxypeptidase-like 35 (LOC109325435)	0.0889	0.1150	0.5574	0.2686	0.0183	0.2225	0.5818	0.6650	0.2509	0.0289	white
XM_019569643.1	serine carboxypeptidase-like 40 (LOC109334067)	0.0899	0.0568	0.6066	0.2657	0.0188	0.2579	0.4409	0.8006	0.4083	0.0253	white
XM_019595298.1	serine carboxypeptidase-like 40 (LOC109353123)	0.0956	0.0645	0.6041	0.2949	0.0186	0.2650	0.3772	0.6700	0.3224	0.0415	white
XM_019583084.1	serine carboxypeptidase-like 42 (LOC109344338)	0.0731	0.1147	0.6099	0.2146	0.0211	0.1121	0.5807	0.6264	0.2305	0.0123	white and Andean
XM_019560770.1	serine carboxypeptidase-like 42 (LOC109327593)	0.0652	0.1616	0.5988	0.2862	0.0333	0.0960	0.6186	0.6405	0.1748	0.0026	Andean
XM_019574179.1	serine carboxypeptidase-like 45 (LOC109337247)	0.1046	0.1029	0.7219	0.3025	0.0175	0.2194	0.4625	0.6884	0.1712	0.0102	white
XM_019574178.1	serine carboxypeptidase-like 45 (LOC109337247)	0.1046	0.1029	0.7219	0.3025	0.0175	0.2194	0.4625	0.6884	0.1712	0.0102	Andean
XM_019587381.1	serine carboxypeptidase-like 45 (LOC109347495)	0.1067	0.1319	0.6587	0.2894	0.0169	0.3608	0.5017	0.5656	0.1557	0.0155	white
XM_019587382.1	serine carboxypeptidase-like 45 (LOC109347495)	0.1267	0.1650	0.5892	0.2861	0.0188	0.3413	0.5030	0.5605	0.1485	0.0195	Andean
XM_019561369.1	serine carboxypeptidase-like 45 (LOC109328097)	0.1073	0.1420	0.3935	0.2292	0.0189	0.1553	0.5722	0.6919	0.2162	0.0254	white and Andean
XM_019596616.1	serine carboxypeptidase-like 45 (LOC109354252)	0.1453	0.1513	0.4064	0.2390	0.0191	0.1729	0.5698	0.6083	0.2399	0.0425	white and Andean
XM_019574554.1	serine carboxypeptidase-like 45 (LOC109337551)	0.1367	0.1514	0.4119	0.3323	0.0196	0.4103	0.5525	0.7367	0.2136	0.0362	Andean

XM_019590295.1	serine carboxypeptidase-like 50 (LOC109349480)	0.1718	0.0997	0.4424	0.2275	0.0391	0.3481	0.6523	0.5771	0.1929	0.0273	white and Andean
XM_019580752.1	serine carboxypeptidase-like 51 (LOC109342765)	0.1248	0.1101	0.4616	0.2146	0.0291	0.1679	0.6402	0.7212	0.2243	0.0073	white and Andean
XM_019557789.1	serine carboxypeptidase-like 51 (LOC109325419)	0.0964	0.0856	0.5424	0.2423	0.0301	0.1133	0.6194	0.7315	0.2949	0.0083	white and Andean
XM_019593253.1	serine carboxypeptidase-like 51 (LOC109351694)	0.1091	0.0895	0.6040	0.1976	0.0288	0.1336	0.6432	0.7108	0.3042	0.0058	white and Andean
XM_019598369.1	serine carboxypeptidase-like 51 (LOC109355293)	0.6521	0.3892	0.3906	0.0821	0.2989	0.1327	0.3377	0.1216	0.1932	0.1214	white and Andean
XM_019586212.1	serine carboxypeptidase II-2-like (LOC109346581)	0.1077	0.1043	0.5528	0.3177	0.0285	0.1196	0.4016	0.7548	0.3045	0.0175	white
XM_019598145.1	serine carboxypeptidase II-2-like (LOC109355163)	0.0892	0.1225	0.5631	0.2042	0.0226	0.2018	0.5867	0.7051	0.3287	0.0141	Andean
XM_019601661.1	serine carboxypeptidase II-3-like (LOC109357669)	0.1126	0.1063	0.6329	0.2621	0.0270	0.3101	0.2808	0.8104	0.3815	0.0738	Andean
XM_019602926.1	serine carboxypeptidase II-3-like (LOC109358601)	0.1126	0.1063	0.6329	0.2621	0.0270	0.3101	0.2808	0.8104	0.3815	0.0738	Andean
XM_019588686.1	carboxypeptidase-like (LOC109348303)	0.1323	0.0735	0.5480	0.2103	0.0246	0.3443	0.5690	0.7189	0.2809	0.0218	white and Andean
XM_019566784.1	carboxypeptidase (LOC109331963)	0.1281	0.0826	0.3817	0.1846	0.0260	0.4620	0.5960	0.7080	0.2298	0.0537	white and Andean
XM_019570851.1	probable glutamate carboxypeptidase 2 (LOC109334865)	0.1800	0.1406	0.1460	0.3567	0.0420	0.0226	0.8380	0.5266	0.5878	0.0071	white and Andean
XM_019578633.1	cucumisin-like (LOC109340870)	0.1271	0.1116	0.6604	0.2294	0.0121	0.2048	0.5678	0.5683	0.2036	0.0141	Andean
XM_019559593.1	vignain (LOC109326788)	0.1089	0.1021	0.4286	0.1835	0.0167	0.0805	0.6334	0.6965	0.3466	0.0189	white and Andean
XM_019560779.1	vignain-like (LOC109327604)	0.1257	0.0954	0.5074	0.2046	0.0158	0.0535	0.5999	0.6485	0.3306	0.0249	white and Andean
XM_019561080.1	vacuolar-processing enzyme-like (LOC109327911)	0.1169	0.0829	0.4942	0.1552	0.0348	0.2692	0.5180	0.8423	0.2960	0.0278	white and Andean
XM_019573985.1	vacuolar-processing enzyme-like (LOC109337103)	0.1301	0.0810	0.4796	0.1759	0.0267	0.2104	0.5666	0.7904	0.3643	0.0191	white and Andean
XM_019582316.1	vacuolar-processing enzyme-like (LOC109343833)	0.1088	0.0981	0.5287	0.1547	0.0326	0.2130	0.4667	0.8232	0.3078	0.0259	white and Andean
XM_019603061.1	vacuolar-processing enzyme-like (LOC109358681)	0.1393	0.0781	0.4843	0.1674	0.0235	0.2039	0.5979	0.7627	0.3581	0.0187	white and Andean
XM_019591087.1	legumain (LOC109349992)	0.1113	0.0694	0.4871	0.1811	0.0377	0.2376	0.4665	0.6587	0.4096	0.0302	white and Andean
XM_019587677.1	cysteine proteinase 15A-like (LOC109347683)	0.1449	0.0800	0.5070	0.2118	0.0351	0.2127	0.4250	0.8096	0.3286	0.0076	white and Andean
XM_019602748.1	cysteine proteinase 15A-like (LOC109358491)	0.1386	0.1054	0.4426	0.2194	0.0357	0.3085	0.4234	0.7999	0.2684	0.0041	white and Andean
XM_019591263.1	cysteine proteinase COT44-like (LOC109350120)	0.1292	0.1164	0.4007	0.2433	0.0126	0.0815	0.4589	0.7109	0.2749	0.0041	white and Andean
XM_019581527.1	aspartic proteinase-like (LOC109343296)	0.1126	0.0637	0.4864	0.1694	0.0262	0.1110	0.4161	0.8013	0.3992	0.0116	white and Andean
XM_019574378.1	aspartic proteinase-like (LOC109337407)	0.1156	0.0645	0.4243	0.1396	0.0286	0.1968	0.4634	0.7888	0.4477	0.0121	white
XM_019585091.1	aspartic proteinase-like (LOC109345851)	0.0998	0.0664	0.5300	0.1627	0.0360	0.3128	0.4293	0.6557	0.3714	0.0115	white
XM_019585092.1	aspartic proteinase-like (LOC109345851)	0.0998	0.0664	0.5300	0.1627	0.0360	0.3128	0.4293	0.6557	0.3714	0.0115	Andean
XM_019572888.1	aspartic proteinase-like (LOC109336349)	0.1319	0.0532	0.4971	0.1910	0.0337	0.0869	0.4848	0.6309	0.4178	0.0093	white
XM_019572890.1	aspartic proteinase-like (LOC109336349)	0.1319	0.0532	0.4971	0.1910	0.0337	0.0869	0.4848	0.6309	0.4178	0.0093	Andean
XM_019563716.1	aspartic proteinase-like (LOC109329853)	0.0902	0.0543	0.4846	0.1421	0.0338	0.2676	0.4675	0.6521	0.3871	0.0085	white
XM_019570715.1	aspartic proteinase-like (LOC109334777)	0.1143	0.0661	0.4530	0.1043	0.0304	0.2612	0.4355	0.7543	0.4356	0.0206	white
XM_019579380.1	aspartic proteinase-like (LOC109341462)	0.1126	0.0637	0.4864	0.1694	0.0262	0.1110	0.4161	0.8013	0.3992	0.0116	Andean
XM_019596747.1	aspartic proteinase-like (LOC109354330)	0.1182	0.0570	0.5316	0.1716	0.0388	0.1037	0.4668	0.5836	0.3765	0.0116	Andean
XM_019582061.1	aspartic proteinase-like protein 1 (LOC109343653)	0.0997	0.1024	0.2775	0.6500	0.0232	0.1403	0.3355	0.3731	0.2534	0.0298	white and Andean
XM_019601373.1	aspartic proteinase-like protein 1 (LOC109357417)	0.0853	0.0787	0.3883	0.6058	0.0304	0.1763	0.3113	0.3550	0.3248	0.0432	white
XM_019601372.1	aspartic proteinase-like protein 1 (LOC109357417)	0.0858	0.0783	0.3908	0.6050	0.0308	0.1746	0.3114	0.3544	0.3284	0.0434	Andean
XM_019572203.1	aspartic proteinase-like protein 2 (LOC109335927)	0.0750	0.0562	0.2769	0.6949	0.0280	0.0936	0.4325	0.4849	0.3589	0.0426	white and Andean
XM_019576096.1	aspartic proteinase-like protein 2 (LOC109338782)	0.1124	0.0567	0.3999	0.5850	0.0202	0.1144	0.5300	0.6287	0.4236	0.0399	white and Andean
XM_019592491.1	aspartic proteinase-like protein 2 (LOC109351129)	0.0708	0.0966	0.3975	0.5326	0.0218	0.2359	0.3436	0.4612	0.3436	0.0626	white and Andean
XM_019596627.1	aspartic proteinase-like protein 2 (LOC109354261)	0.0909	0.0819	0.2878	0.6110	0.0236	0.0903	0.4572	0.5005	0.3806	0.0306	white and Andean
XM_019597169.1	aspartic proteinase-like protein 2 (LOC109354564)	0.0863	0.0717	0.2670	0.7234	0.0247	0.0555	0.3300	0.5146	0.2841	0.0233	white and Andean
XM_019593261.1	aspartic proteinase-like protein 2 (LOC109351698)	0.0739	0.0817	0.2808	0.6144	0.0207	0.0852	0.3608	0.4980	0.3383	0.0474	white and Andean
XM_019597174.1	aspartic proteinase-like protein 2 (LOC109354566)	0.1259	0.0916	0.6492	0.2644	0.0211	0.1641	0.5349	0.6808	0.2652	0.0083	white and Andean
XM_019600038.1	aspartic proteinase-like protein 2 (LOC109356628)	0.0768	0.0737	0.2644	0.6030	0.0349	0.1786	0.4672	0.4498	0.2985	0.0506	white and Andean

XM_019597173.1	aspartic proteinase-like protein 2 (LOC109354565)	0.0778	0.0691	0.2676	0.6835	0.0232	0.0772	0.3643	0.5081	0.2847	0.0512	white and Andean
XM_019591304.1	aspartic proteinase-like protein 2 (LOC109350150)	0.2010	0.0753	0.2172	0.7549	0.0231	0.0647	0.3255	0.7175	0.5351	0.0727	white and Andean
XM_019604031.1	aspartic proteinase-like protein 2 (LOC109359386)	0.5034	0.3456	0.6263	0.3314	0.0715	0.1021	0.2219	0.1182	0.0430	0.0181	white and Andean
XM_019605507.1	aspartic proteinase-like protein 2 (LOC109360538)	0.2471	0.0955	0.3468	0.5708	0.0235	0.1309	0.6336	0.3446	0.2293	0.4899	white and Andean
XM_019580764.1	aspartic proteinase-like protein 2 (LOC109342774)	0.0685	0.0914	0.3511	0.6710	0.0234	0.1264	0.3319	0.4333	0.2876	0.0441	white and Andean
XM_019587876.1	aspartic proteinase-like protein 2 (LOC109347803)	0.0868	0.0693	0.3450	0.6802	0.0243	0.0955	0.4119	0.4091	0.2453	0.0635	white and Andean
XM_019584334.1	aspartic proteinase-like protein 2 (LOC109345371)	0.0781	0.0578	0.3207	0.6764	0.0259	0.1301	0.4151	0.4489	0.3643	0.0478	white and Andean
XM_019575454.1	aspartic proteinase-like protein 2 (LOC109338257)	0.0762	0.1150	0.1982	0.7407	0.0223	0.2309	0.2819	0.4860	0.3891	0.1540	white
XM_019569273.1	aspartic proteinase-like protein 2 (LOC109333715)	0.0872	0.0853	0.3483	0.6402	0.0166	0.1449	0.3769	0.5249	0.3617	0.0748	white
XM_019596247.1	aspartic proteinase-like protein 2 (LOC109353886)	0.4019	0.2538	0.6643	0.1098	0.1018	0.2551	0.3376	0.0927	0.0692	0.2282	white
XM_019587871.1	aspartic proteinase-like protein 2 (LOC109347798)	0.0835	0.0736	0.3199	0.6688	0.0229	0.1094	0.3802	0.4456	0.2528	0.0448	Andean
XM_019587875.1	aspartic proteinase-like protein 2 (LOC109347799)	0.0854	0.0756	0.3539	0.5089	0.0264	0.1708	0.5358	0.5099	0.2952	0.0503	Andean
XM_019575453.1	aspartic proteinase-like protein 2 (LOC109338257)	0.0767	0.1135	0.1963	0.7403	0.0223	0.2285	0.2808	0.4898	0.3922	0.1547	Andean
XM_019596128.1	aspartic proteinase-like protein 2 (LOC109353760)	0.4369	0.4361	0.6872	0.1435	0.1085	0.0546	0.1585	0.1799	0.0602	0.0187	Andean
XM_019600594.1	aspartic proteinase Asp1 (LOC109356941)	0.1133	0.1102	0.4581	0.2357	0.0351	0.3617	0.5155	0.4636	0.1983	0.0093	white
XM_019600595.1	aspartic proteinase Asp1 (LOC109356941)	0.1167	0.1014	0.4901	0.2242	0.0320	0.3365	0.4666	0.4847	0.1797	0.0074	Andean
XM_019603087.1	aspartic proteinase Asp1-like (LOC109358693)	0.1049	0.1243	0.5067	0.2779	0.0391	0.2278	0.4073	0.5922	0.3000	0.0150	white and Andean
XM_019562072.1	aspartic proteinase Asp1-like (LOC109328557)	0.1191	0.0771	0.4949	0.2667	0.0341	0.1837	0.5151	0.5565	0.3785	0.0154	white
XM_019559804.1	leucine aminopeptidase 1-like (LOC109326907)	0.1351	0.0897	0.0466	0.0817	0.3483	0.9737	0.1066	0.0786	0.0465	0.0141	white and Andean
XM_019577002.1	leucine aminopeptidase 1-like (LOC109339545)	0.1167	0.0770	0.0356	0.0736	0.3568	0.9724	0.0900	0.0690	0.0553	0.0146	white and Andean
XM_019586007.1	leucine aminopeptidase 1-like (LOC109346442)	0.6964	0.2010	0.0389	0.1182	0.4805	0.6164	0.1240	0.0909	0.0563	0.1976	white and Andean
XM_019597923.1	leucine aminopeptidase 1-like (LOC109355021)	0.7179	0.2038	0.0267	0.1202	0.5235	0.5248	0.1344	0.0876	0.0554	0.2801	white and Andean
XM_019563474.1	cathepsin B-like (LOC109329719)	0.1299	0.1058	0.5183	0.1561	0.0255	0.4691	0.4735	0.5462	0.2398	0.0101	white and Andean
XM_019566841.1	cathepsin B-like (LOC109332004)	0.1199	0.0892	0.4655	0.2516	0.0278	0.5711	0.4721	0.5700	0.2941	0.0076	white and Andean
XM_019600316.1	subtilisin-like protease Glyma18g48580 (LOC109356781)	0.0911	0.0959	0.6762	0.2434	0.0202	0.1240	0.4308	0.5810	0.1806	0.0095	white and Andean
XM_019604689.1	subtilisin-like protease Glyma18g48580 (LOC109359979)	0.0802	0.1129	0.6148	0.2558	0.0255	0.1458	0.3399	0.4533	0.2376	0.0215	white
XM_019596114.1	subtilisin-like protease Glyma18g48580 (LOC109353749)	0.0830	0.1145	0.6354	0.2317	0.0315	0.1485	0.3259	0.4728	0.2536	0.0266	Andean
XM_019604687.1	subtilisin-like protease Glyma18g48580 (LOC109359979)	0.0802	0.1129	0.6148	0.2558	0.0255	0.1458	0.3399	0.4533	0.2376	0.0215	Andean
XM_019609684.1	subtilisin-like protease Glyma18g48580 (LOC109363425)	0.0984	0.0981	0.6219	0.2143	0.0227	0.1244	0.4670	0.4807	0.2759	0.0087	Andean
XM_019606037.1	subtilisin-like protease SBT1.1 (LOC109360859)	0.1107	0.1220	0.6320	0.2130	0.0158	0.1685	0.4771	0.5637	0.2543	0.0064	white and Andean
XM_019589799.1	subtilisin-like protease SBT1.2 (LOC109349116)	0.1273	0.1070	0.6421	0.2150	0.0185	0.1783	0.4165	0.4935	0.2307	0.0098	white
XM_019591135.1	subtilisin-like protease SBT1.2 (LOC109350027)	0.1519	0.0996	0.6380	0.2196	0.0164	0.1844	0.4887	0.5653	0.3071	0.0094	white
XM_019558537.1	subtilisin-like protease SBT1.3 (LOC109325922)	0.1300	0.1200	0.5568	0.2260	0.0250	0.1647	0.5619	0.5065	0.2517	0.0138	white
XM_019597285.1	subtilisin-like protease SBT1.3 (LOC109354646)	0.1132	0.1432	0.6177	0.2190	0.0184	0.1976	0.5038	0.5535	0.2444	0.0111	white and Andean
XM_019573468.1	subtilisin-like protease SBT1.3 (LOC109336711)	0.1341	0.1286	0.6057	0.2047	0.0224	0.2410	0.4952	0.5140	0.2012	0.0102	Andean
XM_019572380.1	subtilisin-like protease SBT1.4 (LOC109336036)	0.1522	0.1252	0.4799	0.1811	0.0184	0.1611	0.5806	0.5030	0.1951	0.0138	white and Andean
XM_019605527.1	subtilisin-like protease SBT1.5 (LOC109360555)	0.1940	0.1194	0.4519	0.2556	0.0307	0.1823	0.6067	0.5919	0.1805	0.0127	white and Andean
XM_019568543.1	subtilisin-like protease SBT1.5 (LOC109333202)	0.1019	0.1348	0.7317	0.2373	0.0154	0.1831	0.4705	0.4655	0.1851	0.0338	white
XM_019568544.1	subtilisin-like protease SBT1.5 (LOC109333202)	0.1019	0.1348	0.7317	0.2373	0.0154	0.1831	0.4705	0.4655	0.1851	0.0338	Andean
XM_019557444.1	subtilisin-like protease SBT1.6 (LOC109325231)	0.1589	0.1086	0.5407	0.2234	0.0254	0.1837	0.5353	0.5364	0.1930	0.0134	white and Andean
XM_019581372.1	subtilisin-like protease SBT1.7 (LOC109343185)	0.1086	0.0937	0.7110	0.2599	0.0191	0.1411	0.3886	0.5254	0.2516	0.0095	white and Andean
XM_019582359.1	subtilisin-like protease SBT1.7 (LOC109343865)	0.0982	0.1323	0.6828	0.2234	0.0139	0.2135	0.3519	0.5069	0.1924	0.0190	white and Andean
XM_019567712.1	subtilisin-like protease SBT1.7 (LOC109332687)	0.1327	0.1730	0.5541	0.2249	0.0210	0.2270	0.4372	0.4644	0.1725	0.0088	white and Andean
XM_019576301.1	subtilisin-like protease SBT1.7 (LOC109338954)	0.1201	0.0961	0.6721	0.2827	0.0159	0.0961	0.4713	0.5734	0.2992	0.0105	white and Andean

XM_019576964.1	subtilisin-like protease SBT1.8 (LOC109339508)	0.1350	0.1267	0.5104	0.2597	0.0154	0.2319	0.6328	0.5892	0.2273	0.0108	white and Andean
XM_019599648.1	subtilisin-like protease SBT1.8 (LOC109356319)	0.1237	0.0896	0.5749	0.2090	0.0139	0.2718	0.6352	0.5496	0.2310	0.0276	white and Andean
XM_019600892.1	subtilisin-like protease SBT1.9 (LOC109357141)	0.1155	0.1217	0.5820	0.2309	0.0253	0.1435	0.5710	0.4927	0.2153	0.0316	white and Andean
XM_019560236.1	subtilisin-like protease SBT2.2 (LOC109327169)	0.1625	0.1296	0.6244	0.2049	0.0299	0.1762	0.4998	0.6209	0.2099	0.0072	white and Andean
XM_019561928.1	subtilisin-like protease SBT2.3 (LOC109328457)	0.1462	0.1264	0.6118	0.2103	0.0234	0.1047	0.4760	0.5842	0.2712	0.0092	white and Andean
XM_019604449.1	subtilisin-like protease SBT2.3 (LOC109359753)	0.1768	0.1500	0.5606	0.1854	0.0243	0.1511	0.4616	0.5684	0.2063	0.0086	white and Andean
XM_019599085.1	subtilisin-like protease SBT2.5 (LOC109355843)	0.1732	0.1518	0.7341	0.2573	0.0374	0.2089	0.4649	0.6518	0.3003	0.0117	white
XM_019599102.1	subtilisin-like protease SBT2.5 (LOC109355843)	0.1732	0.1518	0.7341	0.2573	0.0374	0.2089	0.4649	0.6518	0.3003	0.0117	Andean
XM_019594317.1	subtilisin-like protease SBT2.5 (LOC109352361)	0.1386	0.1065	0.7209	0.2497	0.0315	0.1327	0.4413	0.6579	0.3536	0.0079	white
XM_019594319.1	subtilisin-like protease SBT2.5 (LOC109352361)	0.1386	0.1065	0.7209	0.2497	0.0315	0.1327	0.4413	0.6579	0.3536	0.0079	Andean
XM_019586948.1	subtilisin-like protease SBT2.6 (LOC109347238)	0.2104	0.1517	0.7305	0.2894	0.0437	0.1880	0.3986	0.6139	0.3320	0.0130	white
XM_019586955.1	subtilisin-like protease SBT2.6 (LOC109347238)	0.2104	0.1517	0.7305	0.2894	0.0437	0.1880	0.3986	0.6139	0.3320	0.0130	Andean
XM_019603621.1	subtilisin-like protease SBT3.17 (LOC109359098)	0.1945	0.1581	0.6199	0.1657	0.0286	0.3200	0.4298	0.5809	0.2320	0.0099	white and Andean
XM_019603971.1	subtilisin-like protease SBT3.17 (LOC109359345)	0.2246	0.1263	0.5130	0.1467	0.0318	0.2596	0.5435	0.4877	0.2794	0.0063	white and Andean
XM_019589598.1	subtilisin-like protease SBT3.9 (LOC109348971)	0.1202	0.1339	0.6700	0.2530	0.0212	0.2037	0.4793	0.4849	0.2095	0.0445	white and Andean
XM_019565461.1	subtilisin-like protease SBT3.9 (LOC109331146)	0.1162	0.1487	0.6553	0.2704	0.0183	0.0875	0.4823	0.4734	0.2363	0.0408	white and Andean
XM_019608751.1	subtilisin-like protease SBT3.9 (LOC109362745)	0.2298	0.3633	0.6990	0.3153	0.0822	0.2376	0.3164	0.2818	0.1466	0.1062	white
XM_019608750.1	subtilisin-like protease SBT3.9 (LOC109362745)	0.2298	0.3633	0.6990	0.3153	0.0822	0.2376	0.3164	0.2818	0.1466	0.1062	Andean
XM_019578615.1	subtilisin-like protease SBT4.14 (LOC109340853)	0.1415	0.1298	0.5434	0.2672	0.0166	0.1770	0.3915	0.4718	0.2188	0.0201	Andean
XM_019606378.1	subtilisin-like protease SBT5.3 (LOC109361072)	0.1238	0.1064	0.6677	0.2444	0.0153	0.1058	0.5143	0.5196	0.2179	0.0119	white and Andean
XM_019557733.1	subtilisin-like protease SBT5.4 (LOC109325370)	0.1013	0.1441	0.6474	0.2339	0.0148	0.0955	0.3757	0.4233	0.1154	0.0088	Andean
XM_019564352.1	subtilisin-like protease SBT5.6 (LOC109330243)	0.1544	0.1218	0.5572	0.2214	0.0250	0.2706	0.5697	0.5054	0.1708	0.0059	white and Andean
XM_019566210.1	subtilisin-like protease SBT6.1 (LOC109331606)	0.1866	0.1675	0.1075	0.3563	0.0767	0.0996	0.7619	0.4994	0.5061	0.0277	white and Andean
