# Supplementary material for: Asparagine-Guided Regulation of Redox Status and Autophagy in Sugar-Starved Lupin (Lupinus spp.) Embryonic Axes—A Transcriptomic and Proteomic Approach
Source: Int J Mol Sci. 2026 Jul 30;27(15):6851. doi: 10.3390/ijms27156851 (PMC13467101; doi:10.3390/ijms27156851)
Supplement: Supplementary file 1 [file ijms-27-06851-s001.zip › Supplementary Table S6 - Subcellular localization - hydrolases.pdf]

Supplementary Table S6. Prediction of subcellular localization of **hydrolases** in white and Andean lupin embryonic axes. DeepLoc 2.0 software (https://services.healthtech.dtu.dk/services/DeepLoc-2.0/) with a high-quality model was used. A localization is predicted if its probability is above the threshold value - green filling of a cell. Blue cell filling indicates the best result, but it is below the threshold.

Gene ID	Name	Cytoplasm	Nucleus	Extra-cellular	Cell membrane	Mitochon-drion	Plastid	Endoplasmic reticulum	**Vacuole**	Golgi apparatus	Peroxisome	Lupin species
	**Probability threshold**	**0.4761**	**0.5014**	**0.6173**	**0.5646**	**0.6220**	**0.6395**	**0.6090**	**0.5848**	**0.6494**	**0.7364**	
XM_019598846.1	purple acid phosphatase-like (LOC109355607)	0.0925	0.0827	0.6406	0.3399	0.0163	0.3001	0.4801	0.6860	0.3855	0.0046	white and Andean
XM_019576218.1	purple acid phosphatase-like (LOC109338868)	0.1510	0.0782	0.5512	0.3375	0.0168	0.1974	0.4851	0.7017	0.4457	0.0063	Andean
XM_019576228.1	purple acid phosphatase-like (LOC109338879)	0.1570	0.0858	0.6005	0.3511	0.0144	0.1972	0.4447	0.6879	0.4172	0.0065	Andean
XM_019582485.1	purple acid phosphatase 2-like (LOC109343952)	0.1120	0.1315	0.5921	0.2712	0.0187	0.3392	0.4485	0.6275	0.4654	0.0093	white
XM_019576128.1	purple acid phosphatase 3-like (LOC109338808)	0.1244	0.1244	0.5847	0.3349	0.0681	0.2140	0.5247	0.5879	0.3542	0.0124	Andean
XM_019608887.1	purple acid phosphatase 7-like (LOC109362842)	0.1583	0.1041	0.5607	0.1885	0.0371	0.2663	0.5876	0.6047	0.3368	0.0072	white and Andean
XM_019594544.1	purple acid phosphatase 15 (LOC109352529)	0.1724	0.1101	0.4170	0.3119	0.0202	0.2883	0.4764	0.6994	0.3238	0.0263	white and Andean
XM_019576127.1	purple acid phosphatase 17-like (LOC109338807)	0.1601	0.1237	0.5423	0.2655	0.0499	0.1442	0.6385	0.5045	0.4035	0.0049	white and Andean
XM_019574800.1	purple acid phosphatase 18-like (LOC109337759)	0.0992	0.1078	0.6281	0.2189	0.0190	0.3547	0.4350	0.7298	0.2851	0.0187	white and Andean
XM_019579961.1	purple acid phosphatase 18-like (LOC109342000),	0.1209	0.0869	0.5422	0.2078	0.0213	0.2677	0.3947	0.7370	0.3300	0.0235	white
XM_019579954.1	purple acid phosphatase 18-like (LOC109342000)	0.1209	0.0869	0.5422	0.2078	0.0213	0.2677	0.3947	0.7370	0.3300	0.0235	Andean
XM_019569323.1	probable purple acid phosphatase 20 (LOC109333757)	0.1104	0.1023	0.7143	0.2789	0.0159	0.2711	0.4674	0.7004	0.3382	0.0132	white and Andean
XM_019592069.1	probable purple acid phosphatase 20 (LOC109350770)	0.0955	0.1070	0.7281	0.2680	0.0244	0.2827	0.5235	0.6557	0.3768	0.0129	Andean
XM_019576654.1	probable purple acid phosphatase 20 (LOC109339241)	0.0911	0.0996	0.7576	0.2576	0.0205	0.3064	0.5086	0.6384	0.3801	0.0151	Andean
XM_019598955.1	purple acid phosphatase 22-like (LOC109355750)	0.1483	0.1108	0.4534	0.2040	0.0244	0.1715	0.6960	0.6064	0.3425	0.0179	white and Andean
XM_019595680.1	purple acid phosphatase 23 (LOC109353416)	0.1080	0.0794	0.2163	0.6813	0.0208	0.1272	0.4564	0.6092	0.5231	0.0535	white and Andean
XM_019585182.1	bifunctional purple acid phosphatase 26 (LOC109345908)	0.1051	0.1027	0.6101	0.2952	0.0213	0.3883	0.4051	0.5119	0.3204	0.0365	white and Andean
XM_019566487.1	probable inactive purple acid phosphatase 1 (LOC109331775)	0.1334	0.1108	0.6562	0.2782	0.0148	0.1500	0.3535	0.5306	0.2882	0.0185	white and Andean
XM_019595313.1	probable inactive purple acid phosphatase 1 (LOC109353137)	0.1662	0.1588	0.4071	0.2417	0.0215	0.1687	0.4779	0.5648	0.4106	0.0094	white and Andean
XM_019598938.1	probable inactive purple acid phosphatase 1 (LOC109355737)	0.1453	0.1327	0.6048	0.2491	0.0211	0.1688	0.4057	0.5693	0.3229	0.0177	white
XM_019566064.1	probable inactive purple acid phosphatase 2 (LOC109331520)	0.1705	0.1150	0.2658	0.4124	0.0300	0.2086	0.6013	0.5595	0.4134	0.0369	white and Andean
XM_019571243.1	probable inactive purple acid phosphatase 16 (LOC109335156)	0.1543	0.1513	0.4281	0.2137	0.0386	0.2879	0.4233	0.6299	0.3946	0.0098	white
XM_019571244.1	probable inactive purple acid phosphatase 16 (LOC109335156)	0.1582	0.1443	0.4029	0.2420	0.0323	0.2418	0.4389	0.5803	0.3196	0.0094	Andean
XM_019580811.1	probable inactive purple acid phosphatase 27 (LOC109342803)	0.1750	0.1137	0.5214	0.2845	0.0182	0.1506	0.5379	0.5410	0.3118	0.0085	white
XM_019609744.1	probable inactive purple acid phosphatase 27 (LOC109363480)	0.1374	0.1326	0.5211	0.2302	0.0328	0.3029	0.4665	0.4516	0.3873	0.0175	white
XM_019606920.1	probable inactive purple acid phosphatase 27 (LOC109361461)	0.3875	0.2708	0.5223	0.7034	0.1296	0.1544	0.2436	0.1930	0.2013	0.0459	Andean
XM_019575594.1	probable inactive purple acid phosphatase 28 (LOC109338376)	0.2097	0.2007	0.2266	0.1860	0.0781	0.1757	0.7512	0.6183	0.6848	0.0115	white and Andean
XM_019563171.1	probable inactive purple acid phosphatase 29 (LOC109329489)	0.4542	0.3749	0.3408	0.2434	0.3433	0.1184	0.5316	0.3325	0.2093	0.0172	white and Andean
XM_019584931.1	probable inactive purple acid phosphatase 29 (LOC109345746)	0.1947	0.1864	0.4281	0.2021	0.0370	0.3685	0.5746	0.4490	0.1928	0.0056	white and Andean
XM_019606347.1	probable inactive purple acid phosphatase 29 (LOC109361048)	0.1357	0.1253	0.4989	0.1761	0.0305	0.3335	0.5015	0.4580	0.2848	0.0056	white and Andean
XM_019572759.1	phosphatidate phosphatase PAH1-like (LOC109336264)	0.4622	0.2427	0.0370	0.3484	0.2596	0.1078	0.4697	0.6589	0.6137	0.0714	white
XM_019572761.1	phosphatidate phosphatase PAH1-like (LOC109336264)	0.4647	0.2423	0.0364	0.3495	0.2644	0.1054	0.4715	0.6595	0.6114	0.0707	Andean
XM_019606101.1	phosphatidate phosphatase PAH1-like (LOC109360901)	0.4662	0.2388	0.0362	0.3432	0.2657	0.1097	0.4671	0.6675	0.6105	0.0636	white
XM_019606105.1	phosphatidate phosphatase PAH1-like (LOC109360901)	0.4702	0.2364	0.0370	0.3427	0.2706	0.1084	0.4756	0.6667	0.6126	0.0626	Andean
XM_019575183.1	phosphatidate phosphatase PAH2-like (LOC109338047)	0.4672	0.2199	0.0422	0.4362	0.2926	0.0942	0.4269	0.5069	0.5402	0.0910	white and Andean
XM_019582400.1	phosphatidate phosphatase PAH2-like (LOC109343889)	0.4979	0.2444	0.0364	0.4803	0.2076	0.1337	0.5037	0.6096	0.6003	0.1390	white

XM_019582401.1	phosphatidate phosphatase PAH2-like (LOC109343889)	0.4979	0.2444	0.0364	0.4803	0.2076	0.1337	0.5037	0.6096	0.6003	0.1390	Andean
XM_019565331.1	alpha-mannosidase-like (LOC109331057)	0.1156	0.0678	0.4894	0.1808	0.0235	0.2222	0.4889	0.5772	0.3363	0.0131	white and Andean
XM_019572185.1	alpha-mannosidase 2-like (LOC109335920)	0.1802	0.1842	0.0890	0.1808	0.0566	0.0441	0.4689	0.5316	0.8611	0.0209	white and Andean
XM_019587054.1	alpha-mannosidase 2-like (LOC109347307)	0.1834	0.1943	0.0853	0.1532	0.0556	0.0369	0.4998	0.5814	0.8645	0.0195	white
XM_019587055.1	alpha-mannosidase 2-like (LOC109347307)	0.1834	0.1943	0.0853	0.1532	0.0556	0.0369	0.4998	0.5814	0.8645	0.0195	Andean
XM_019587058.1	alpha-mannosidase 2-like (LOC109347309)	0.1834	0.1943	0.0853	0.1532	0.0556	0.0369	0.4998	0.5814	0.8645	0.0195	white
XM_019591864.1	probable alpha-mannosidase At5g13980 (LOC109350642)	0.1652	0.0707	0.5154	0.2465	0.0199	0.1622	0.5610	0.7153	0.3236	0.0102	white and Andean
XM_019574565.1	probable alpha-mannosidase At5g13980 (LOC109337557)	0.1455	0.1305	0.4867	0.2315	0.0309	0.1646	0.5250	0.6962	0.3772	0.0192	white and Andean
XM_019591865.1	alpha-mannosidase At3g26720-like (LOC109350643)	0.1417	0.0758	0.6189	0.2951	0.0356	0.1079	0.5399	0.6293	0.3526	0.0066	white
XM_019591871.1	alpha-mannosidase At3g26720-like (LOC109350643)	0.1298	0.0701	0.6089	0.3089	0.0314	0.0911	0.5365	0.6497	0.3512	0.0048	Andean
XM_019571389.1	mannosyl-oligosaccharide 1,2-alpha-mannosidase MNS1-like (LOC109335257)	0.1162	0.2320	0.1224	0.1138	0.0433	0.0262	0.5516	0.4020	0.8485	0.0909	white and Andean
XM_019600840.1	mannosyl-oligosaccharide 1,2-alpha-mannosidase MNS1-like (LOC109357110)	0.1203	0.2226	0.1367	0.1274	0.0559	0.0221	0.5850	0.3531	0.8292	0.0963	white and Andean
XM_019608869.1	mannosyl-oligosaccharide 1,2-alpha-mannosidase MNS1-like (LOC109362834)	0.1375	0.1928	0.1356	0.1223	0.0591	0.0121	0.6127	0.3833	0.8666	0.1104	white and Andean
XM_019587164.1	mannosyl-oligosaccharide 1,2-alpha-mannosidase MNS1-like (LOC109347365)	0.1373	0.2114	0.1239	0.0967	0.0586	0.0073	0.5995	0.3701	0.8917	0.1075	white
XM_019584157.1	mannosyl-oligosaccharide 1,2-alpha-mannosidase MNS3 (LOC109345269)	0.1418	0.1951	0.1274	0.1016	0.0700	0.0073	0.6483	0.2658	0.8374	0.0620	white and Andean
XM_019573088.1	alpha-mannosidase I MNS4 (LOC109336475)	0.1309	0.0815	0.2949	0.2432	0.0405	0.0477	0.6460	0.5613	0.4677	0.0384	white and Andean
XM_019601077.1	alpha-mannosidase I MNS5 (LOC109357251)	0.2035	0.1017	0.3101	0.2135	0.0475	0.0148	0.6823	0.6196	0.5909	0.0264	white
XM_019601086.1	alpha-mannosidase I MNS5 (LOC109357251)	0.3903	0.1822	0.3823	0.3290	0.2082	0.0496	0.4925	0.3923	0.5485	0.3472	Andean
XM_019603348.1	beta-amylase-like (LOC109358869)	0.1510	0.1412	0.0748	0.1719	0.1160	0.8639	0.2361	0.3039	0.3940	0.1909	white and Andean
XM_019573943.1	beta-amylase 7 (LOC109337068)	0.3033	0.6900	0.0987	0.2041	0.0766	0.0573	0.1246	0.1847	0.1110	0.1178	white and Andean
XM_019567791.1	beta-amylase 8 (LOC109332766)	0.3605	0.7029	0.0668	0.2059	0.0640	0.0537	0.1230	0.1589	0.0959	0.0326	white and Andean
XM_019570531.1	inactive beta-amylase 9-like (LOC109334651)	0.1047	0.1487	0.0146	0.0655	0.1299	0.9572	0.0593	0.1009	0.1364	0.0308	white and Andean
XM_019584173.1	inactive beta-amylase 9-like (LOC109345278)	0.0960	0.1278	0.0214	0.0626	0.1222	0.9505	0.0639	0.1095	0.1530	0.0306	white and Andean
XM_019563548.1	inactive beta-amylase 9-like (LOC109329756)	0.0955	0.1328	0.0223	0.0583	0.1469	0.9234	0.0725	0.0985	0.1763	0.0429	white
XM_019578853.1	acid beta-fructofuranosidase-like (LOC109341057)	0.1268	0.1273	0.3309	0.3090	0.0382	0.0380	0.5053	0.7274	0.8381	0.0448	white and Andean
XM_019589524.1	acid beta-fructofuranosidase-like (LOC109348914)	0.1260	0.1143	0.3617	0.5556	0.0222	0.0432	0.4676	0.8045	0.6959	0.0293	White
XM_019591143.1	acid beta-fructofuranosidase-like (LOC109350031)	0.1345	0.1193	0.3301	0.3047	0.0471	0.0439	0.5848	0.7507	0.8559	0.1261	Andean
XM_019591144.1	acid beta-fructofuranosidase-like (LOC109350031)	0.1339	0.1186	0.3958	0.2778	0.0574	0.0402	0.5443	0.6768	0.8664	0.0926	
XM_019558626.1	beta-fructofuranosidase, soluble isoenzyme I-like (LOC109325976)	0.1217	0.1340	0.2781	0.3459	0.0360	0.0997	0.4789	0.8515	0.7180	0.0932	white
XM_019577363.1	beta-fructofuranosidase, insoluble isoenzyme 1-like (LOC109339841)	0.0768	0.0856	0.5142	0.2616	0.0321	0.2361	0.7011	0.6868	0.3518	0.0095	Andean
XM_019587567.1	beta-fructofuranosidase, insoluble isoenzyme CWINV3-like (LOC109347628)	0.4257	0.2240	0.6218	0.3965	0.0504	0.1334	0.4114	0.1414	0.0922	0.0845	white
XM_019587566.1	beta-fructofuranosidase, insoluble isoenzyme CWINV3-like (LOC109347628)	0.1954	0.1111	0.5203	0.2590	0.0409	0.2964	0.3764	0.5145	0.2433	0.0596	Andean
XM_019601938.1	beta-fructofuranosidase, insoluble isoenzyme CWINV6-like (LOC109357882)	0.5940	0.3535	0.2809	0.2381	0.0550	0.0349	0.2858	0.4437	0.2719	0.3874	Andean
XM_019608208.1	beta-glucosidase 11-like (LOC109362424)	0.1195	0.0700	0.6086	0.2918	0.0322	0.1007	0.4520	0.6024	0.1126	0.0216	white and Andean
XM_019608209.1	beta-glucosidase 11-like (LOC109362425)	0.1095	0.0682	0.5882	0.2827	0.0325	0.1014	0.4519	0.5620	0.0938	0.0173	white and Andean

XM_019557531.1	beta-glucosidase 11-like (LOC109325270)	0.1311	0.0741	0.5284	0.3310	0.0328	0.0959	0.5454	0.5969	0.1181	0.0228	white and Andean
XM_019605021.1	beta-glucosidase 11-like (LOC109360269)	0.1316	0.0762	0.4752	0.2888	0.0429	0.1076	0.5433	0.5248	0.1230	0.0110	white
XM_019605024.1	beta-glucosidase 11-like (LOC109360269)	0.1308	0.0753	0.4949	0.2899	0.0429	0.1074	0.5405	0.5357	0.1174	0.0094	Andean
XM_019558600.1	beta-glucosidase 12-like (LOC109325961)	0.5779	0.2296	0.5842	0.2389	0.2157	0.2389	0.2288	0.0971	0.1482	0.0570	white and Andean
XM_019601597.1	beta-glucosidase 12-like (LOC109357626)	0.1083	0.0670	0.6591	0.2221	0.0234	0.2070	0.5137	0.6893	0.1744	0.0040	white and Andean
XM_019592741.1	beta-glucosidase 12-like (LOC109351302)	0.0871	0.0781	0.6106	0.2276	0.0294	0.3686	0.4950	0.6188	0.1422	0.0045	white
XM_019577581.1	beta-glucosidase 13-like (LOC109340022)	0.0912	0.0609	0.5470	0.3123	0.0325	0.2570	0.3936	0.5662	0.0930	0.0111	white
XM_019569753.1	beta-glucosidase 18-like (LOC109334162)	0.1768	0.1356	0.4108	0.2723	0.0341	0.1621	0.4163	0.6838	0.2157	0.0405	white and Andean
XM_019569755.1	beta-glucosidase 18-like (LOC109334164)	0.1626	0.1151	0.4566	0.3163	0.0253	0.1955	0.4340	0.6924	0.1992	0.0177	white
XM_019565010.1	beta-glucosidase 40-like (LOC109330729)	0.0976	0.0771	0.6212	0.2518	0.0223	0.2496	0.3843	0.6072	0.0984	0.0133	white
XM_019565129.1	beta-glucosidase 40-like (LOC109330856)	0.5439	0.3101	0.2123	0.3118	0.1147	0.0993	0.1671	0.2195	0.1249	0.0369	white and Andean
XM_019568629.1	beta-glucosidase 40-like (LOC109333250)	0.5491	0.2546	0.4308	0.3519	0.1962	0.4535	0.2482	0.2508	0.0805	0.1465	white
XM_019568625.1	beta-glucosidase 40-like (LOC109333249)	0.1017	0.0706	0.6162	0.2641	0.0262	0.3239	0.3361	0.5865	0.0715	0.0243	white
XM_019590359.1	beta-glucosidase 40-like (LOC109349529)	0.1312	0.0701	0.5273	0.2733	0.0378	0.1304	0.5574	0.6188	0.1526	0.0168	white and Andean
XM_019565304.1	beta-glucosidase 40-like (LOC109331021)	0.1313	0.0881	0.6131	0.2649	0.0221	0.1497	0.4143	0.6660	0.1134	0.0112	Andean
XM_019593824.1	putative beta-glucosidase 41 (LOC109352044)	0.1272	0.0769	0.6343	0.3456	0.0243	0.1854	0.4704	0.6167	0.1482	0.0477	white and Andean
XM_019600527.1	beta-glucosidase 42 (LOC109356901)	0.7157	0.4917	0.0893	0.1486	0.1326	0.2942	0.3967	0.3595	0.1331	0.1361	white
XM_019603084.1	beta-glucosidase 44-like (LOC109358694)	0.1283	0.0803	0.5240	0.3254	0.0295	0.3472	0.4731	0.6332	0.1703	0.0236	white and Andean
XM_019595085.1	beta-glucosidase 46-like (LOC109352899)	0.1189	0.0880	0.6349	0.3053	0.0329	0.2203	0.3372	0.5953	0.0980	0.0216	white
XM_019572067.1	beta-glucosidase BoGH3B-like (LOC109335846)	0.0669	0.0779	0.6014	0.2291	0.0108	0.0362	0.3754	0.5715	0.1567	0.0073	white
XM_019572069.1	beta-glucosidase BoGH3B-like (LOC109335847)	0.0956	0.0823	0.6839	0.2905	0.0107	0.0509	0.3157	0.6368	0.2008	0.0122	white
XM_019574569.1	beta-glucosidase BoGH3B-like (LOC109337560)	0.6991	0.3312	0.2410	0.1263	0.0728	0.2871	0.2117	0.1894	0.0922	0.3522	white
XM_019564443.1	beta-glucosidase BoGH3B-like (LOC109330295)	0.0546	0.0495	0.2598	0.6232	0.0174	0.0312	0.4558	0.4453	0.2581	0.0753	white
XM_019575720.1	beta-glucosidase BoGH3B-like (LOC109338466)	0.0529	0.0477	0.2883	0.6766	0.0173	0.0278	0.4015	0.5144	0.3230	0.0457	white
XM_019597527.1	beta-glucosidase BoGH3B-like (LOC109354794)	0.0696	0.0713	0.6816	0.2944	0.0108	0.0405	0.3216	0.6124	0.1601	0.0086	white
XM_019602793.1	alpha-galactosidase-like (LOC109358516)	0.0741	0.1055	0.5527	0.1400	0.0227	0.3220	0.2559	0.5949	0.3302	0.0304	white
XM_019602794.1	alpha-galactosidase-like (LOC109358516)	0.0732	0.1056	0.5624	0.1420	0.0229	0.3119	0.2521	0.5943	0.3275	0.0308	Andean
XM_019557239.1	alpha-galactosidase 1-like (LOC109325093)	0.0818	0.0792	0.6149	0.2863	0.0256	0.2656	0.3017	0.5755	0.2701	0.0652	Andean
XM_019594856.1	alpha-galactosidase 1-like (LOC109352690)	0.0836	0.0961	0.5323	0.2473	0.0349	0.3264	0.3198	0.6233	0.2589	0.0420	white and Andean
XM_019581713.1	alpha-galactosidase 3 (LOC109343439)	0.1160	0.0919	0.5027	0.2128	0.0349	0.5224	0.5168	0.8188	0.3265	0.0566	white and Andean
XM_019608643.1	probable alpha-galactosidase B (LOC109362678)	0.1279	0.1276	0.5084	0.2481	0.0389	0.1601	0.5846	0.5882	0.3648	0.0074	white
XM_019608642.1	probable alpha-galactosidase B (LOC109362678)	0.1292	0.1246	0.5139	0.2489	0.0396	0.1589	0.5786	0.5875	0.3674	0.0076	Andean
XM_019587836.1	phospholipase D alpha 1 (LOC109347779)	0.5800	0.1841	0.1748	0.6032	0.1683	0.2697	0.4506	0.6470	0.2899	0.0169	white and Andean
XM_019558767.1	phospholipase D alpha 1-like (LOC109326104)	0.5371	0.1540	0.1385	0.6605	0.1621	0.2392	0.4059	0.6394	0.2463	0.0323	white
XM_019558955.1	phospholipase D alpha 1-like (LOC109326249)	0.1279	0.1276	0.5084	0.2481	0.0389	0.1601	0.5846	0.5882	0.3648	0.0074	white
XM_019561209.1	phospholipase D alpha 1-like (LOC109327993)	0.5536	0.1685	0.1247	0.6465	0.1623	0.2502	0.4210	0.6096	0.2289	0.0281	white and Andean
XM_019589661.1	phospholipase D alpha 1-like (LOC109349027)	0.5741	0.1763	0.1758	0.6100	0.1623	0.2398	0.4600	0.6571	0.2811	0.0222	white and Andean
XM_019592546.1	phospholipase D alpha 1-like (LOC109351174)	0.5536	0.1747	0.1857	0.6204	0.1798	0.2564	0.4394	0.6764	0.2519	0.0270	white and Andean
XM_019600872.1	phospholipase D alpha 1-like (LOC109357128)	0.6130	0.2020	0.1866	0.5862	0.1801	0.3616	0.4372	0.5999	0.2007	0.0269	white and Andean
XM_019601391.1	phospholipase D alpha 4-like (LOC109357430)	0.5712	0.1616	0.1744	0.6508	0.2209	0.2770	0.4054	0.6318	0.3456	0.0145	white and Andean
XM_019580943.1	phospholipase D alpha 4 (LOC109342897)	0.5345	0.1486	0.1801	0.6625	0.2090	0.2895	0.4129	0.6629	0.4055	0.0097	white and Andean
XM_019566432.1	phospholipase D beta 1-like (LOC109331745)	0.5322	0.2214	0.1758	0.6728	0.2185	0.2861	0.4727	0.5999	0.2172	0.0425	white
XM_019566431.1	phospholipase D beta 1-like (LOC109331745)	0.4566	0.2805	0.2567	0.5015	0.2001	0.5883	0.4624	0.4625	0.2355	0.0524	Andean
XM_019578403.1	phospholipase D gamma 1-like (LOC109340688)	0.5999	0.2679	0.0899	0.5087	0.0923	0.1678	0.4928	0.5374	0.3121	0.0654	white

XM_019578405.1	phospholipase D gamma 1-like (LOC109340688)	0.5976	0.2629	0.1111	0.5448	0.1160	0.2136	0.4948	0.5338	0.3189	0.0714	Andean
XM_019605735.1	phospholipase D gamma 1-like (LOC109360683)	0.5972	0.2875	0.0967	0.4745	0.0990	0.0913	0.5285	0.5087	0.2240	0.0701	white and Andean
XM_019582536.1	phospholipase D delta-like (LOC109343985)	0.5972	0.2875	0.0967	0.4745	0.0990	0.0913	0.5285	0.5087	0.2240	0.0701	white and Andean
XM_019589314.1	phospholipase D delta-like (LOC109348766)	0.5167	0.1763	0.1603	0.7060	0.1986	0.1982	0.4278	0.7166	0.4251	0.0301	white and Andean
XM_019566398.1	phospholipase D delta-like (LOC109331721)	0.5157	0.1830	0.1739	0.7090	0.2027	0.2327	0.4265	0.7292	0.4350	0.0300	white and Andean
XM_019563488.1	phospholipase D zeta 1 (LOC109329727)	0.5151	0.1307	0.1128	0.5334	0.1877	0.2042	0.4119	0.7890	0.4835	0.0126	white and Andean
XM_019597723.1	phospholipase D zeta 1-like (LOC109354912)	0.5649	0.1242	0.1236	0.4993	0.2056	0.1932	0.3967	0.8090	0.4580	0.0181	white and Andean
XM_019609101.1	phospholipase D zeta 1-like (LOC109362978)	0.5290	0.1168	0.1320	0.5316	0.2066	0.2236	0.3926	0.8099	0.4905	0.0187	white and Andean
XM_019582257.1	phospholipase D Z-like (LOC109343782)	0.2293	0.0692	0.5601	0.2356	0.0788	0.0184	0.5885	0.5938	0.2938	0.0019	white and Andean
XM_019576285.1	glycerophosphodiester phosphodiesterase GDPD2-like (LOC109338933)	0.4327	0.2836	0.0737	0.1272	0.2486	0.8046	0.1296	0.1253	0.1285	0.0530	white and Andean
XM_019562049.1	glycerophosphodiester phosphodiesterase GDPDL3-like (LOC109328540)	0.1578	0.1091	0.6412	0.3418	0.0192	0.1952	0.3529	0.5704	0.2439	0.0087	white
XM_019605437.1	glycerophosphodiester phosphodiesterase GDPDL3-like (LOC109360493)	0.0987	0.0568	0.4617	0.5901	0.0231	0.1294	0.4218	0.6331	0.3733	0.0102	white and Andean
XM_019580108.1	glycerophosphodiester phosphodiesterase GDPDL3-like (LOC109342097)	0.1067	0.0539	0.4218	0.6139	0.0184	0.1280	0.4181	0.5613	0.3429	0.0099	white and Andean
XM_019579045.1	glycerophosphodiester phosphodiesterase GDPD4 (LOC109341197)	0.2247	0.0783	0.4059	0.2461	0.2079	0.2452	0.7182	0.4387	0.5073	0.0213	white and Andean
XM_019583354.1	glycerophosphodiester phosphodiesterase GDPD6 (LOC109344588)	0.1180	0.0470	0.5561	0.2420	0.0529	0.3374	0.5181	0.7137	0.2895	0.0090	white
XM_019583357.1	glycerophosphodiester phosphodiesterase GDPD6 (LOC109344588)	0.1180	0.0470	0.5561	0.2420	0.0529	0.3374	0.5181	0.7137	0.2895	0.0090	Andean
XM_019607470.1	glycerophosphodiester phosphodiesterase GDPD6-like (LOC109361945)	0.0925	0.0704	0.4600	0.2112	0.0324	0.2948	0.4478	0.6356	0.2145	0.0156	white and Andean
XM_019609050.1	glycerophosphodiester phosphodiesterase GDPDL7-like (LOC109362947)	0.0904	0.0562	0.6205	0.6992	0.0259	0.1481	0.3101	0.6211	0.4167	0.0152	white and Andean
XM_019597793.1	glycerophosphodiester phosphodiesterase GDPDL7-like (LOC109354947)	0.0927	0.0621	0.6252	0.6552	0.0204	0.1342	0.2622	0.6249	0.4122	0.0163	white and Andean
XM_019559589.1	glycerophosphodiester phosphodiesterase GDPDL7-like (LOC109326786)	0.1163	0.0595	0.5847	0.6490	0.0210	0.0934	0.3551	0.6434	0.3390	0.0115	white
XM_019585741.1	glycerophosphodiester phosphodiesterase GDPDL7-like (LOC109346268)	0.1012	0.0562	0.5743	0.6538	0.0222	0.1128	0.3101	0.6824	0.4301	0.0157	white
XM_019597226.1	acidic endochitinase-like (LOC109354612)	0.1081	0.1011	0.6913	0.2102	0.0198	0.2518	0.4605	0.5026	0.2439	0.0138	white and Andean
XM_019597230.1	acidic endochitinase-like (LOC109354613)	0.0956	0.0930	0.7585	0.2073	0.0189	0.4134	0.4688	0.5896	0.1929	0.0229	white
XM_019597229.1	acidic endochitinase-like (LOC109354613)	0.0956	0.0930	0.7585	0.2073	0.0189	0.4134	0.4688	0.5896	0.1929	0.0229	Andean
XM_019586547.1	acidic endochitinase-like (LOC109346772)	0.0852	0.0921	0.8435	0.1680	0.0114	0.1809	0.2971	0.4824	0.1518	0.0045	white and Andean
XM_019558790.1	endochitinase PR4 (LOC109326121)	0.0896	0.1016	0.8154	0.1385	0.0159	0.1899	0.2182	0.4311	0.1446	0.0085	white and Andean
XM_019561272.1	endochitinase A2 (LOC109328029)	0.0785	0.0978	0.8313	0.2180	0.0094	0.2668	0.2334	0.4755	0.1180	0.0458	white and Andean
XM_019596629.1	chitinase-like protein 1 (LOC109354262)	0.1488	0.0793	0.7182	0.2118	0.0213	0.3575	0.4790	0.5964	0.1840	0.0056	white and Andean
XM_019564050.1	chitinase-like protein 1 (LOC109330059)	0.1567	0.0813	0.7830	0.1943	0.0296	0.5515	0.4238	0.5702	0.2078	0.0102	white and Andean
XM_019584635.1	chitinase-like protein 2 (LOC109345559)	0.1060	0.0763	0.9097	0.1463	0.0216	0.2782	0.3247	0.6411	0.2433	0.0040	white
XM_019584633.1	chitinase-like protein 2 (LOC109345559)	0.1060	0.0763	0.9097	0.1463	0.0216	0.2782	0.3247	0.6411	0.2433	0.0040	Andean
XM_019564265.1	chitinase-like protein 2 (LOC109330193)	0.1187	0.0866	0.8833	0.1887	0.0222	0.4039	0.3428	0.6234	0.2501	0.0116	white and Andean
XM_019574242.1	chitinase 2-like (LOC109337298)	0.7015	0.3349	0.3590	0.2613	0.2028	0.0344	0.2403	0.1530	0.2977	0.1093	white and Andean

XM_019608203.1	chitinase-3-like protein 1 (LOC109362420)	0.1235	0.0787	0.8505	0.2120	0.0167	0.5303	0.3894	0.6023	0.1544	0.0106	white and Andean
XM_019595909.1	chitinase 10-like (LOC109353573)	0.0953	0.0995	0.6312	0.1767	0.0171	0.2683	0.3472	0.3552	0.1600	0.0073	white and Andean
XM_019560766.1	chitinase a-like (LOC109327590)	0.1675	0.0828	0.8540	0.2671	0.0172	0.6835	0.5151	0.4194	0.2070	0.0224	white and Andean
XM_019585666.1	chitinase a-like (LOC109346226)	0.0898	0.0801	0.8773	0.1791	0.0149	0.2829	0.3936	0.4892	0.1588	0.0112	white and Andean
XM_019592030.1	chitinase a-like (LOC109350742)	0.1553	0.1152	0.6945	0.1839	0.0162	0.3603	0.3265	0.5079	0.1860	0.0152	white and Andean
