# Supplementary material for: Asparagine-Guided Regulation of Redox Status and Autophagy in Sugar-Starved Lupin (Lupinus spp.) Embryonic Axes—A Transcriptomic and Proteomic Approach
Source: Int J Mol Sci. 2026 Jul 30;27(15):6851. doi: 10.3390/ijms27156851 (PMC13467101; doi:10.3390/ijms27156851)
Supplement: Supplementary file 1 [file ijms-27-06851-s001.zip › Supplementary Table S8 - Primer sequences.pdf]

Supplementary Table S8. Primer sequences used for qRT-PCR reactions. Actin was used as an endogenous control.

Gene ID	Forward primer (5'-3')	Reverse primer (5'-3')	Amplicon length (bp)
White lupin			
XM_019565749.1	tggctacctgttggttaaaatgc	tcgtctgagctttcatcacc	126
XM_019581579.1	gatttttgaattggggtgattgcg	tcttcaccttttcccaccatgg	120
XM_019563162.1	cacctcaagggttggctga	cctcccggtcactagttgg	137
XM_019559989.1	acttgccattagaacctcaatcc	acattcaaccctgcaaatgac	139
XM_019572924.1	ctcgccaagatgcgaacatc	caaccgaaatcccatcggg	132
XM_019593765.1	ctgaggacccggttgttctg	tggattgaggtgcagcgttg	137
XM_019591263.1	gcatatgaagcccgcgatag	caactgcagccactgttgag	124
XM_019559593.1	ggcataacagcagaaagcag	cgcagtgcgtcttcgttg	131
XM_019591864.1	actcacttttctttggccgg	ggaacgcaccagcaaagatc	129
XM_019578853.1	agggacatgatacattggcaac	ccgagtcattggttgaacccg	145
XM_019603084.1	cccaaatggaactggcgaag	ctcaagagccaaagggagg	130
XM_019605437.1	tcagctcagagatctaacaccag	cgaaaccataggcatcctcac	124
XM_019592546.1	gagcctcagagatgttgggag	ctcctcccggtttagacctc	133
XM_019608887.1	acaatgtagatcactctggctg	gcctacctatgcatgcatgg	128
XM_019585272.1 (actin)	ctcctgctatgtatgttgctatac	gagagcataaccttcatagatagg	
Andean lupin			
XM_019565749.1	ggatcaattggctacctgctg	atcgtcagagctttcatcacc	135
XM_019581579.1	aagagacttttgatttggggtg	cgtcacccttttccaccatg	122
XM_019563162.1	tctctcacacccgaagacttg	tggctttccggtcactagttg	147
XM_019559989.1	ggtggtgtaacagcatatggag	gctccacaaggaagatgagg	149
XM_019581527.1	ggcagctctaatctctgggtcc	catactgaattgcagcacgag	130
XM_019593765.1	gattctcctgctggtgtcgg	cgggttggaaagaaactccg	138
XM_019591263.1	tgactaatgaggaatttcgtgcc	cactggtaacctatcacgggc	122
XM_019559593.1	gcaccccgagtggtaacag	ccaacaactaccacatcgacc	125
XM_019591864.1	gaggagtttggtgtgactcc	tgcgcttagccctatcttgg	145
XM_019578853.1	tcagaaacagtaatggagaatctg	acataggaccgttaggatcg	123
XM_019603084.1	agagcgtcgttcccaaagg	cccctgggttacggtgaatg	127
XM_019592546.1	aggagttactaaactttatgcgacc	tgccatatgagcacaataaatgtg	130
XM_019608887.1	ctttggcacaatctgaggc	ttgccttaaggatgggaacaag	120
XM_019598846.1	ggtttacaacccctccccc	acatagagcacggtctgcc	142
XM_019585272.1 (actin)	ctccggctatgtatgttgcc	gagagcataaccttcgtagatagg	
